# A Computer-Based Interactive Narrative and a Serious Game for Children With Asthma: Development and Content Validity Analysis

**DOI:** 10.2196/28796

**Published:** 2021-09-13

**Authors:** Made Ary Sarasmita, Luh Putu Febryana Larasanty, Li-Na Kuo, Kuei-Ju Cheng, Hsiang-Yin Chen

**Affiliations:** 1 Department of Clinical Pharmacy School of Pharmacy Taipei Medical University Taipei Taiwan; 2 Program Study of Pharmacy Faculty of Mathematics and Science Udayana University Badung Indonesia; 3 Department of Pharmacy Taipei Medical University Wan Fang Hospital Taipei Taiwan

**Keywords:** asthma, computer, children, interactive narrative, serious game, digital education, mobile phone

## Abstract

**Background:**

Nonadherence to medications, failure to prevent exposure to asthma triggers, lack of knowledge about using medications, and fixed mindsets contribute to poor asthma control in children. Digital learning could provide a new strategy for improving health-related outcomes in children with asthma.

**Objective:**

The aim of this study is to develop and design a digital educational program, titled Module of Inhaler and Asthma Triggers for Children (MIRACLE), for Indonesian children with asthma. The program comprises an interactive narrative and a serious game. It was proposed to increase the understanding of asthma self-management, instruct on proper inhaler techniques, improve asthma control, and promote a growth mindset for children with asthma.

**Methods:**

Two phases of research were conducted to develop the program. In the first phase, a literature search and two rounds of the Delphi technique were conducted to obtain agreement from an expert panel regarding elements of asthma self-management and the design of interactive narratives and a serious game. The expert panel item statements were evaluated using the content validity index (CVI). In the second phase, the SERES framework, Norma Engaging Multimedia Design, and Psychological Theory of Growth Mindset were applied to create a storyline, learn objectives, and game challenges.

**Results:**

In the first phase, 40 experts were invited to participate in Delphi round 1. Forty responses were collected to generate 38 item statements that consisted of part 1, elements of asthma self-management (25 items), and part 2, design of an interactive narrative and a serious game (13 items); 38 experts were involved in Delphi round 2. In total, 24 statements in part 1 and 13 items in part 2 had item-CVI values >0.80. The average CVI was 0.9, which was considered acceptable. Four narrative plots and five game sessions were developed during the second phase. Challenges with the scenario, scoring, and feedback on asthma difficulties were designed to promote a growth mindset for learners.

**Conclusions:**

We developed a culture-specific, computer-based asthma program containing an interactive narrative and a serious game to deliver asthma self-management and promote a growth mindset among Indonesian children.

## Introduction

### Background

Designing effective asthma education for children is a challenge for health care professionals, educators, and parents. Insufficient knowledge of asthma and nonadherence to medications in children are associated with high rates of morbidity and mortality [1]. Improved education and support to help pediatric patients manage their disease are urgently recommended to control the burden of asthma [2]. However, frequent occurrences of acute asthma attacks and negative outcomes imply that effective self-management of asthma is difficult to achieve in pediatric patients [3,4]. Children frequently have low engagement with asthma self-care and poor medication adherence [5]. Efforts to develop new asthma self-management education for children must consider their level of understanding and the development of a certain mindset.

Intertwining behavioral stages and a growth mindset in asthma education may help children overcome the challenges of disease exacerbation and engage with learning. Deploying principles from modern behavioral psychology, children with a growth mindset might improve their self-care abilities by identifying impediments and embracing challenges [6]. Children face greater difficulties than older patients in comprehending causal relationships between asthma triggers and their consequences [7]. Previous negative experiences of asthma exacerbations may escalate their fears and discourage them from engaging in asthma self-management activities. An animated web-based interactive brain function program [8] was built to develop a growth mindset and resiliency through interactive games and classroom activities. Children with asthma may be unable to manage their disease because of a lack of concepts [9], and asthma education with a growth mindset might empower children and their families to exercise greater control over their situation. Incorporating a similar approach to promote positive attitudes and behavioral changes may provide new insights into asthma education for children.

Game-based education that incorporates elements of child psychology may encourage children with asthma to acquire knowledge, skills, self-care engagement, and motivation. Serious games were reported to promote positive attitudes and acceptance among children with HIV or AIDS [10], cancer [11], and asthma [12]. A serious game is defined as an entertaining game provided through a digital platform with an enjoyable design, specific rules including challenge goals, and a scoring concept that provides feedback to the player [13]. The well-recognized SERES framework, developed by Verschueren et al [14], provides rigorous scientific and design foundations to inform the design of serious games for health-related outcomes. Using game-based education, children experience opportunities to solve problems through analytical thinking [15] and improve specific skills through training and adequate feedback [16]. Engagement is also essential for programs designed to promote healthy behaviors. Norma Engaging Multimedia Design (NEMD) provides a theory for constructing engaging materials and encouraging thoughts and feelings, and it has been used to develop interactive narratives for asthma education [17].

Appropriate cultural adaptation is an essential element that should be emphasized when creating asthma education for children [18]. A systematic review confirmed that educators should adopt culture-specific values, beliefs, and languages for educational materials to represent real-life role models and social support systems of target populations [19]. Different languages and cultures may act as barriers and decrease the effectiveness of educational programs [20,21]. Indonesia, an archipelagic country with diverse ethnicities, languages, and local beliefs, may require different strategies when delivering asthma education. Culture-specific asthma education proved superior to a generic program in terms of reducing exacerbations requiring hospitalization in children [18]. The development of a serious game related to asthma that adopts Indonesian cultural and language preferences may help fill this gap and strengthen self-management among children with asthma.

### Objective

To address the main issue of delivering effective asthma education to children, we developed a digital asthma educational program titled Module of Inhaler and Asthma Triggers for Children (MIRACLE). It consisted of an interactive narrative, a serious game, and a written asthma action plan. The specific aims of this study were as follows: (1) to conduct the Delphi technique to obtain culture-specific agreements from a variety of relevant stakeholders on the elements and design of asthma education and (2) to design an interactive narrative and a serious game based on the NEMD theory and the SERES framework.

## Methods

### Study Design

**Figure 1:**
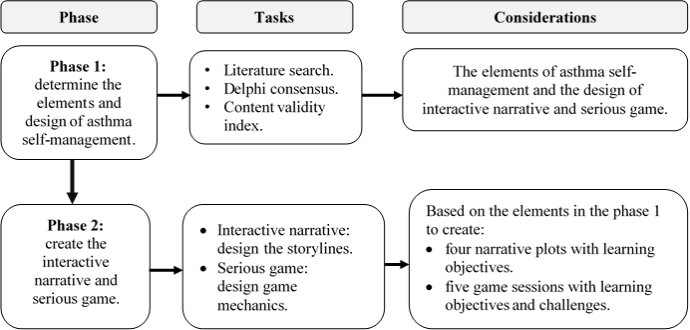
Flowchart of the research phase.

There were two phases in this study, as shown in Figure 1. Phase 1 focused on determining elements of asthma self-management and designing an interactive narrative and serious game using previous literature, a Delphi consensus among experts, and calculation of the content validity index (CVI); phase 2 focused on designing an interactive narrative and a serious game using the NEMD theory and SERES framework. Ethical approval for this study was obtained from the Ethics Committee of the Faculty of Medicine, Udayana University, Indonesia (no. 2020.03.1.0865). Health care professionals who agreed to participate in this study received information on the study on the web and provided informed consent before the Delphi consensus was initiated.

### Phase 1: Determining the Elements and Design of Asthma Self-management

#### Delphi Expert Panel

The expert panel consisted of physicians and clinical pharmacists invited to develop the elements and design an interactive narrative and serious game [22]. We attempted to invite diverse categories of health care providers from academia, hospital care, and primary care to accurately capture the content required for asthma information in various clinical settings in Indonesia. Physicians who served on the expert panel specialized in pediatrics and had practiced pediatric ambulatory care for patients with asthma for at least 1 year. Clinical pharmacists had at least a master’s degree and had practiced in pediatric care or pediatric research for at least 2 years. After providing written informed consent, panelists were invited to participate in the panel every week via reminders sent by WhatsApp Messenger (Facebook) with a link to a Google Forms survey (Google). The researchers conducted a web-based consensus among health care providers to identify all categories of expected needs for Indonesian children with asthma via a two-round modified Delphi study. Participants answered questions and provided initial judgments until agreement was obtained using an anonymous, iterative technique to avoid the influence of different perspectives [23]. Individual responses were neither excluded nor shared with the other panelists. The Delphi study was conducted from May to July 2020.

In the first round of the Delphi process, researchers sent an open question to the experts using Google Forms: “According to your experience, what are the components of an interactive narrative and serious game that children can understand about asthma?” All suggestions collected in the first round were used to generate structural item statements. These statements were then categorized into two parts, including elements of asthma self-management (part 1) and the design of an interactive narrative and serious game (part 2). Generated statements were constructed with a four-point Likert scale (1=very irrelevant, 2=irrelevant, 3=relevant, and 4=very relevant) and sent to the experts again for Delphi round 2.

#### Content Validation

After the second round of Delphi, we used the CVI to quantify the content validity of the item statements. It relies on expert ratings for each item based on the relevance of asthma self-management and the design of the interactive narrative and serious game. Item-specific CVI (I-CVI) was also calculated for each item statement by counting the number of experts who rated the item as 3 (relevant) or 4 (very relevant) and dividing that number by the total number of experts [24]. The overall instrument-CVI was defined *as* “the average proportion of items rated as 3 or 4 (valid) across the experts,” as the average CVI. It was calculated by summing the I-CVIs and dividing them by the number of items. We used a CVI cutoff point of at least 80% (a minimum I-CVI of 0.80) as acceptable [25] and an average CVI of ≥0.90 to have excellent content validity [26]. The experts were also prompted to comment on and justify their responses.

### Phase 2: Creating an Interactive Narrative and Serious Game

#### General Design

After agreement was achieved, the elements and design identified in the two Delphi rounds were used to create an interactive narrative and serious game with the assistance of a multimedia expert. A serious game was created to support children’s growth mindset after learning about asthma self-management through an interactive narrative. The main considerations included designing the storylines, characters, and learning objectives in the interactive narrative, and designing the game mechanics, learning objectives, and challenges in the serious game. Cartoon characters were created to reflect the cultural preferences of Indonesian children.

#### Target Audience

The appropriate age range for the target audience to receive this program was children aged 6-12 years based on their cognitive development and need for asthma education [27]. Researchers invited 1 pediatrician and 2 clinical pharmacists to provide feedback on the design of the interactive narrative and serious game. The proposed sequences of using this computer-based asthma educational intervention were that learners would engage with the interactive narrative and then play a serious game.

#### Design of the Interactive Narrative

##### Scientific Foundation

The design of the interactive narrative applied the NEMD theory and transformed the information we sought to convey into storylines with learning objectives. It was visualized using Articulate Storyline, version 2.0 (Articulate Global). This approach allows a designer to create a nonlinear storyline for which branches of the story differ, but each plot shares the same trunk of characters and learning objectives of asthma self-management. It allows the target audience to select actions and cartoon characters. The storyline adopted the NEMD theory, including simulation interactivity, construct interactivity, and immediacy.

##### Design Foundations

Sketches of cartoon characters were illustrated by a pharmacist using the Sketchbook Draw and Paint app, downloaded from the Google Android Play Store (Autodesk), and the sketches were imported into Articulate Storyline. The voices of pharmacists delivering asthma information and music were recorded and embedded in the software. The software generated HTML5-compatible output, which could be accessed on a PC.

#### Design of the Serious Game

##### Scientific Foundation

The scientific foundations of the SERES framework were applied to develop the serious game. The target audience, the theoretical basis from the literature, and the CVI were assessed using the iterative Delphi method. The learning objectives of the serious game were to achieve active learning of asthma self-management and increase confidence and the growth mindset of children with asthma.

##### Design Foundations

The design foundations included learning mechanics, game mechanics, and design requirements. Learning mechanics were defined according to the learning objectives [28], and these were changed into game mechanics, which incorporated the concept of a growth mindset. The concept of a growth mindset was incorporated into game sessions by providing challenges and rewarding efforts. Design requirements considered the platform and display appropriate to the children’s mindset and their level of understanding. The user interface of the serious game was developed using the Construct 2D game programming software, version 2.0 (Scirra) and run on a Windows desktop platform. Several characteristics of using Construct 2.0, in this study, allowed researchers to publish the game on desktop computers (PC or Mac [Apple Inc]), Android mobile platforms, and websites via HTML5, relying on its event system and requiring no programming language or coding experience. The technical details of Construct 2.0 included a game design template, game objects, event system such as execution of commands, fun factors such as challenges and rewards, and the game genre. Game flow explained how the game was played from the beginning until a player either won or lost.

## Results

### Phase 1: Determining the Elements of Asthma Self-management

Of the 50 experts invited with various areas of expertise, including pediatricians and clinical pharmacists, 40 experts agreed to participate in Delphi round 1. The demographic characteristics of the 40 participants are given in Table 1. A total of 40 responses after raising an open question were collected in round 1 (Table S1 in Multimedia Appendix 1).

**Table 1:** Demographic characteristics of the Delphi expert panels.

Characteristic				Round 1 (N=40)		Round 2 (n=38)
Age (years), mean (SD)				33.4 (3.4)		33.3 (3.3)
**Sex,** **n** **(%)**						
	Male		12 (31)		11 (29)	
	Female		28 (69)		27 (71)	
**Health care professional,** **n** **(%)**						
	Physician		15 (38)		14 (38)	
	Pharmacist		25 (63)		24 (63)	
**Specialty,** **n** **(%)**						
	**Physician**					
		Pediatrics	2 (5)		1 (3)	
		Family medicine	7 (18)		7 (18)	
		Other	6 (15)		6 (16)	
	**Pharmacist**					
		Master of Clinical Pharmacy	25 (63)		24 (63)	
**Practice field,** **n** **(%)**						
	Hospital		16 (40)		16 (42)	
	University		20 (50)		18 (47)	
	Primary care		4 (10)		4 (11)	

**Figure 2:**
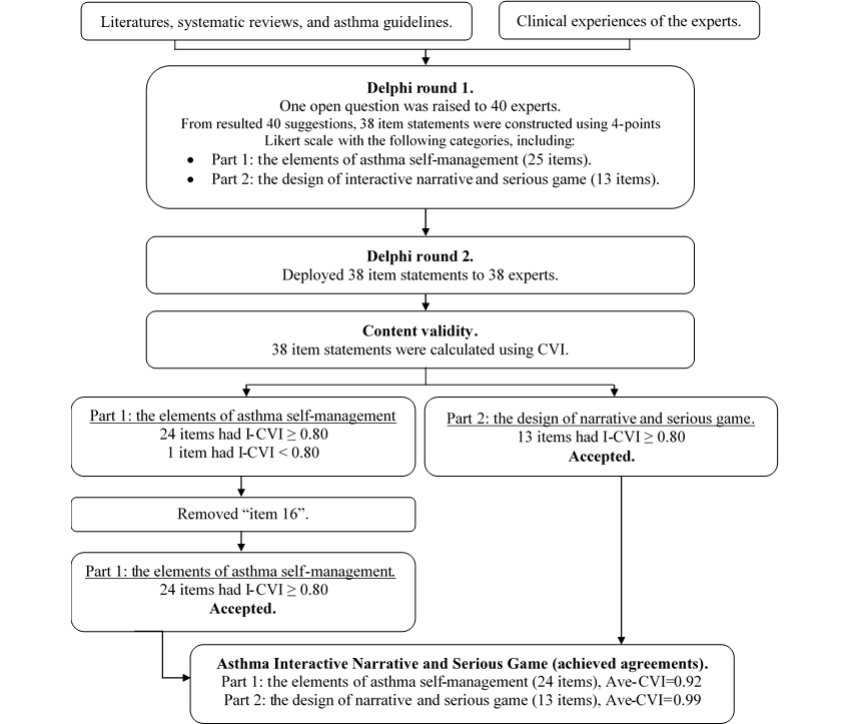
The Delphi process and content validity of the interactive narrative and serious game. Ave-CVI: average content validity index; CVI: content validity index; I-CVI: item content validity index.

Figure 2 demonstrates the Delphi process, including the experts involved in two Delphi rounds to obtain agreement on the elements of asthma self-management and design of the interactive narrative and serious game. Forty suggestions identified in round 1 were summarized into 38 item statements using a four-point Likert scale, including part 1, which consisted of elements of asthma self-management (25 items), and part 2, which consisted of the design (13 items). Two experts declined to participate in Delphi round 2; thus, the 38-item statements were deployed to 38 experts.

Content validity was assessed after the 38 responses were collected, and consisted of the elements of asthma self-management and design of the interactive narrative and serious game (Table S2 in Multimedia Appendix 1). In total, 24 statements in part 1 and 13 item statements in part 2 had I-CVI values of ≥0.8. The experts considered one statement (item 16): (1) “explain the types of inhalers available in Indonesia” to be *not very relevant* or *not relevant*, as this statement had an I-CVI value of 0.76 (<0.80). This item had a low I-CVI value because the experts explained that there were many types of inhalers in Indonesia, which might contribute to confusing the children. According to the panel’s suggestion, this item was removed, and 24 acceptable items on the elements of asthma self-management and 13 acceptable items on the design of the interactive narrative and serious game were retained. The agreement achieved among the experts resulted in average CVI values of 0.92 (for the 24 items of asthma self-management) and 0.99 (for the 13 items of the design), and achieved a universal average CVI of 0.5 (Table S3 in Multimedia Appendix 1). As item 16 was removed, we used a metered-dose inhaler with a spacer in the narrative and game sessions to introduce general information on inhaler use for children.

### Phase 2: Create the Design of the Interactive Narrative and Serious Game

#### Design of the Interactive Narrative

**Figure 3:**
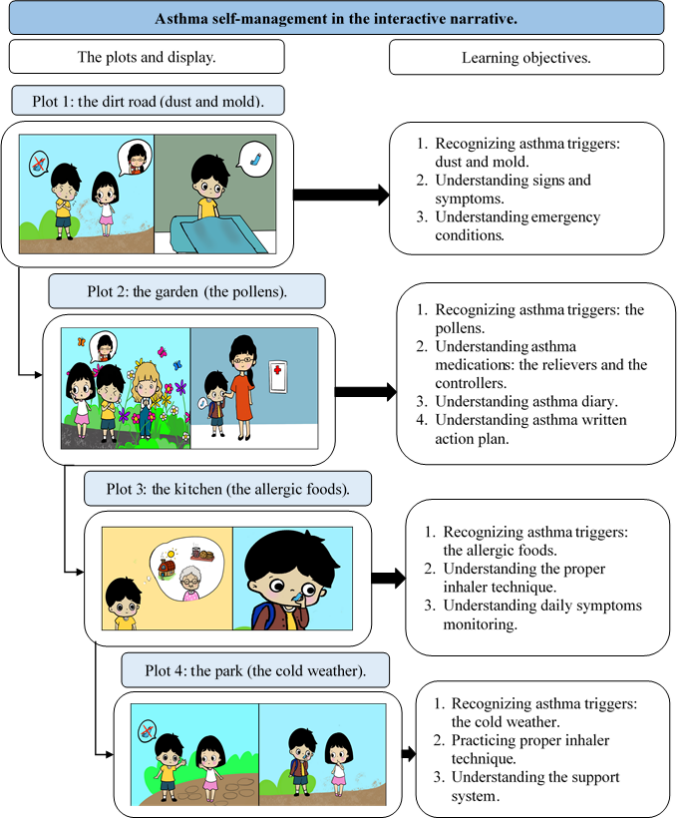
The storyline and the learning objectives of the interactive narrative.

Figure 3 illustrates the plots and learning outcomes of the four storylines. The target audience has to deal with asthma triggers and write an asthma action plan during their cartoon adventure, as structured in the storylines (Figure S3 in Multimedia Appendix 2). Asthma information, purposes, characters, and instructions are provided before the learners engage with the narrative. This combination of strategies contains elements of asthma self-management and supplements each other (Table S1 in Multimedia Appendix 3). Plots were created to represent asthma triggers, including a dirt road (dust and mold), a garden (pollen), a kitchen (food allergies), and a park (cold weather). Plot 1 was created to provide information on asthma triggers encountered in daily activities, asthma signs and symptoms, and emergency conditions and to help children become acquainted with their previous asthma experiences. Plot 2 was concerned with the importance of asthma medications, medication adherence, and a written asthma action plan. Plot 3 was designed to deliver information on proper inhaler techniques and the importance of daily symptom observation. Plot 4 emphasized practicing proper inhaler use and strategies to obtain support from their immediate environment. Each plot contained a different ending to the story and achieved the represented goal by completing the plots without an asthma attack. The learners were asked to repeat the narrative if they failed to achieve the goal. Positive feedback from 3 experts was collected to supplement the design of the narrative (Table S2 in Multimedia Appendix 3).

The visualization and expression of the characters were adjusted for each plot. On the basis of the NEMD theory, construct interactivity describes the availability of activities in the virtual world, as visualized as a short trip to the grandmother’s house. It represents the unique culture of Indonesian children visiting their grandparents in a village during a religious holiday. The cartoon characters were designed as children and families with positive attitudes, and the cartoon family was visualized as gentle and caring to cover simulated interactivity. Learners are able to choose one cartoon character with asthma and engage with that character. Immediacy facilitates learners’ choice and observation of all actions. In each plot, every choice matters, and learners can decide their own options and compromise with all of the resulting choices (Figure S3 in Multimedia Appendix 2). Music and daily conversations among cartoon characters were added to provide fun and active learning.

#### Design of the Serious Game

##### Scientific Foundations

**Figure 4:**
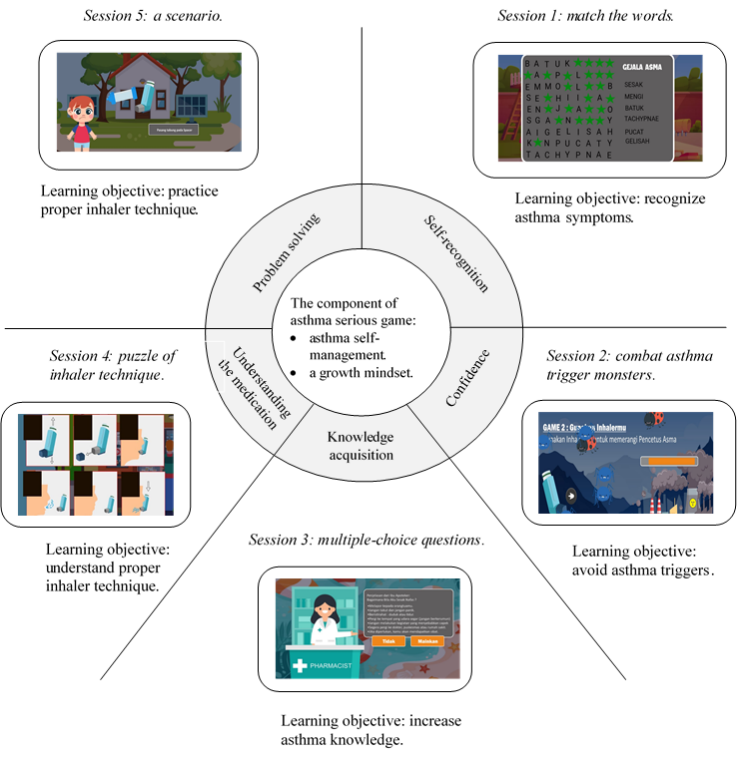
Game sessions, growth mindset, and the learning objectives.

Figure 4 demonstrates a visualization of the serious game with learning objectives, elements of asthma self-management, and the component of a growth mindset. The learning objectives were designed for each session of a serious game. Each session represents the preferences of children as the target audience, such as a cartoon protagonist, a colorful background, and musical tones. Session 1 covers recognizing asthma signs, symptoms, and emergency conditions. Session 2 encourages players to combat asthma trigger monsters. Session 3 was designed to recall knowledge by choosing the correct answer from multiple-choice questions. Session 4 introduces proper inhaler techniques, including how to correctly use, clean, and store the inhaler. Session 5 focuses on practicing steps of correctly using a metered-dose inhaler using a scenario.

##### Design Foundations

Learning mechanics were integrated with the concept of a growth mindset. Before beginning the game, players are able to choose a character to represent themselves in game sessions. Learning mechanics include avoiding skipping content, embedding information through images, and providing feedback. A cartoon pharmacist provides brief information about asthma with an inserted link of images before entering each session to avoid skipping content. The development of a growth mindset is facilitated by ticking asthma difficulties on a checklist and achieving goals in daily life before entering the game sessions. Session 1 was designed to cope with asthma and increase self-recognition. Session 2 aimed to increase self-confidence and reduce fear of exposure to asthma triggers. Sessions 3 and 4 were constructed to improve the understanding of the disease and medications and to achieve goals. Session 5 provides a scenario to help a child use an inhaler with a spacer. After playing sessions 1 and 2, players were asked to write feedback and explain their experiences when experiencing an asthma attack.

The design requirements were transformed into components of the growth mindset, as shown in Figure 4. The visualization and screenshots were designed as a 2D game that facilitated the ability to complete the challenges (Figures S4-S6 in Multimedia Appendix 2). This game presents *lock* badges to reinforce the difficulties and reward efforts required to finish one session before playing the next session. Visual callouts appear to increase excitement in each featured session. The serious game also presents points and stars collected by the players on a score screen after a session is completed. This serious game can be applied directly (offline) on a computer. It is also available on the web as an app that can be downloaded for free and played on any Android PC, smartphone, or tablet (Figure S2 in Multimedia Appendix 2) [29].

**Figure 5:**
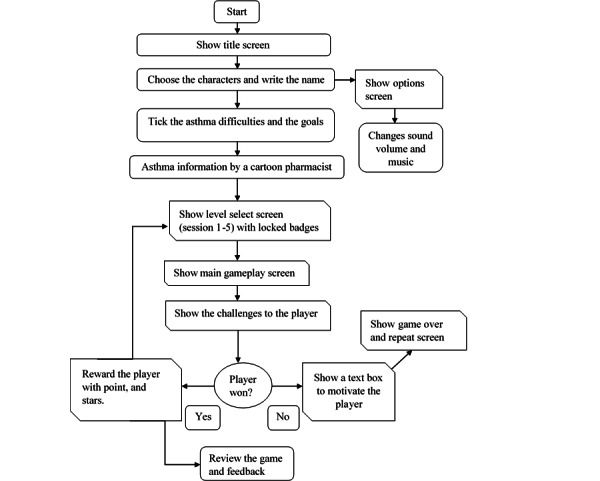
Game flow.

The game design template is described in Table 2, and the game flow is illustrated in Figure 5. To facilitate problem solving for learners with a fixed mindset, game mechanics were explicitly applied in session 5. In session 5, players apply an inhaler with a spacer, which presents the learning mechanics of *selecting an inhaler with a spacer*, *properly applying the inhaler*, *understanding its use*, *and repeating the sequences*.

**Table 2:** Game design template.

Component			Description
Main purpose			Improve asthma self-management and encourage children to act as a manager of their disease
Genre			Casual game with puzzles, questions, and battle
Visual			Cartoon 2D game
**Game mechanics**			
	Session 1	Matching words (making a line)	
	Session 2	Battle (shooting asthma trigger monsters)	
	Session 3	Questions and answers (select an option)	
	Session 4	Puzzle of inhaler (dragging, picking up puzzle pieces, and placing them on the puzzle board)	
	Session 5	A scenario (dragging an inhaler with a spacer)	
**Game behavior**			
	Session 1	Drag and drop, anchor	
	Session 2	Fade, bullet, destroy outside, eight directions	
	Session 3	Drag and drop	
	Session 4	Drag and drop	
	Session 5	Drag and drop, pin	
**Project scope**			
	Number of characters	The player’s character and a cartoon pharmacist	
	Number of sessions	Five sessions	
	Type of enemies	Asthma trigger monsters (game session 2)	
	Type of weapons	Inhaler (game session 2)	
	Number of puzzles	Three puzzles (game session 4)	
Rewards			Point and stars every time a player finishes a session
Growth mindset			Checklist of asthma difficulties Checklist of goals in daily life Writing about asthma experiences and feelings A box with motivational text in each session
Winning condition			Finish each session and unlock the next session
Mastering the program			HTML5, Google Play Store
Image and audio files			Image format: 32-bit PNG^a^ file Audio files: 16-bit PCM^b^ wav files

^a^PNG: Portable Network Graphics.

^b^PCM: pulse code modulation.

## Discussion

### Principal Findings

This study describes the development and design of an Indonesian culture-specific, computer-based asthma education module for children, which comprises an interactive narrative and a serious game. The Delphi consensus was used to gather reliable and relevant perspectives and opinions systematically. Two rounds of a Delphi study and content validity led to low panel attrition and were considered appropriate to achieve agreement [30]. From Delphi rounds 1 and 2, experts emphasized the elements of asthma self-management and the design that should be included in building storylines and game challenges. The panel achieved agreement on 24 items of asthma self-management and 13 items on the design of preferences. The average CVI was acceptable for creating the interactive narrative and serious game. The scientific foundations and expert participatory action employed in this program were designed to overcome the limitations of existing asthma educational tools for children.

According to the 2020 Global Initiative for Asthma guidelines [31], asthma education needs to be delivered at every visit, which confirms that any educational asthma module for children should emphasize the importance of self-management ability. The development of the ability to manage asthma in daily life is highly recommended to enable children to successfully manage their disease. The guideline recommendations for asthma self-management are as follows: (1) identify asthma triggers that may predispose one to an asthma attack, (2) regularly determine an asthma action plan, (3) recognize asthma symptoms, (4) avoid asthma triggers to prevent asthma attacks, and (5) use proper inhaler techniques [27]. Children grow from being dependent to independent adolescents who must manage their asthma on their own; thus, knowledge, growth mindset, and skills of practicing correct inhaler techniques are crucial to asthma educational programs. The same five recommendations listed above were also highlighted during the initial phase of the Delphi technique and were used to develop the story lines and challenges.

The NEMD theory and SERES framework were applied during the second development phase to design the foundations of the module and visualize the interactive narrative and serious game. The interactive narrative presents lessons that are suitable for school-aged children, and the computer-based asthma program relies on the narrative to provide meaning to virtual engagement. Unlike a conventional paper-based story, this study supports active learning in the context of manipulating a plot based on a learner’s decisions. It meets a learner’s needs and expresses thoughts based on their personal asthma experiences. Four plots coherently contained elements of asthma self-management and promoted positive attitudes. Although there was no punishment or reward after learning, achieving these goals is important in daily life.

The SERES framework was applied in several game development studies, which showed that it was effective in reducing learning barriers, such as perioperative anxiety and pain among children [32]. A gamified e-learning module also used the SERES framework to teach the adequate use of personal protective equipment during the COVID-19 pandemic [33]. This study proposes design foundations that encourage children to customize a cartoon character with positive attitudes, as visualized in the 2D journey to finish five sessions with a locked badge. It represents self-representation and is important for increasing their feelings and sense of relating to and engaging with the system. Each visualization in the plots was adjusted to the preferences of children and provided options to deal with each consequence. Take-home messages are offered to encourage children to adhere to asthma therapy and create a written asthma action plan. Our findings also highlighted the importance of achieving goals and providing feedback at the end of each game session and after each narrative.

The unique learning goals and design provided by the interactive narrative and serious game supplement each other, offering exciting opportunities to educate children in solving their daily problems. Several previous games [34,35] taught self-management skills through the recognition of asthma triggers and symptoms and the appropriate use of medications. In this study, the MIRACLE program explicitly covered comprehensive lessons on asthma education, including the pathophysiology, recognition of asthma triggers and symptoms, inhalation techniques, adherence to medications, and compliance. Children usually lack a long attention span and may have a low capacity to perceive terminology, limited coping, and limited social support [36]. A comic-based asthma educational program confirmed that children are highly interested in fun, comprehensive learning games, and comics accompanied by their parents [37]. Narratives in comic visualization also convey illness experiences and reduce feelings of fear [38]. A comprehensive learning tool with an enjoyable design increases user satisfaction, improves perception and acceptance, leads to acquisition of knowledge and skills, and promotes behavioral changes to improve health-related outcomes [39].

This study embedded the theory of cultivating a growth mindset in a computer-based interactive narrative and serious game. Compared with previous games that only included a simple adventure genre [40,41], this serious game encompasses a variety of fun gaming genres, such as adventure, quizzes, puzzles, and a scenario to promote a growth mindset. Asthmatic children face lifelong challenges, and a growth mindset can help them overcome difficulties of the disease and accomplish tasks [42]. Point rewards and badges as part of gamification are essential for attracting children’s attention [43]. A previous study that applied a computer-based neonatal resuscitation training game for nursing students consisted of scenarios with an increasingly difficult neonatal resuscitation activity in each round [44]; it was designed to promote a growth mindset in terms of attitudes and performance. Provision of feedback and challenges in developing a growth mindset are meaningful and relevant to users. The combination of a growth mindset and active learning can enhance children’s self-management skills and the courage to live with asthma.

Generalizability of the MIRACLE program should be a concern because of cultural and language-specific elements and design. Its applicability should be tested in other settings and cultural contexts before direct translation. As language barriers [45] and a fixed mindset seem to contribute to poorer health outcomes and knowledge in children, this study highlighted the importance of developing games using the target users’ own language and promoting a growth mindset by presenting challenges and rewarding efforts. A culturally adapted translation and validation of the elements are needed to address this issue. The concepts and methodology, techniques for obtaining the elements, storylines, and game mechanics can potentially be applied to other clinical research in developing digital-based health education for children in primary school with chronic diseases.

### Limitations

This study did not involve participation by children, and thus, actual children’s feedback on their preferences is lacking. External validity remained unclear because no reliability testing was performed. Despite the fact that the participation rate of medical doctors in the expert panel was 50%, a limited number of pediatricians agreed to participate. However, this study consistently leveraged a wide range of perspectives on asthma self-management. The involvement of primary care doctors and pharmacists is important because of their crucial role in providing information on proper inhaler techniques to patients in clinical and community settings [46]. This computer-based narrative game was developed using an Indonesian cultural context, which in turn influenced the decisions of target audiences in learning asthma self-management and adherence to treatments. Cultural and religious beliefs, health literacy, and life experiences have become limitations in directly translating the MIRACLE program in other countries with different cultural preferences. The Delphi method and content validity may be warranted to adjust the cultural preferences of target audiences to add or delete a few elements of asthma education. Practicing inhaler techniques in this program solely through virtual learning may be inadequate. Supplementing periodic real-time demonstrations and practice by educators may be required to overcome this limitation.

### Further Research

Reliability testing and randomized controlled trials are urgently needed to evaluate the effectiveness of this program and measure the magnitude and retention of changes in health-related outcomes among Indonesian children. The evaluation of satisfaction with the game should be provided after completing all game sessions. Feedback from parents and children could also be acquired after the program was delivered. We propose to evaluate asthma control and quality of life in children using a translated and validated version of the Asthma Control Questionnaire and Pediatric Asthma Quality of Life Questionnaires in Bahasa Indonesia with permission from the original author [47]. Correct inhaler use should be measured using a checklist adapted from the Indonesian Standard of Community Pharmacy Practice [48]. Data before and after the intervention should be measured and evaluated to provide a preliminary overview of the effectiveness of this program among children with asthma. The feedback generated can be used to improve this computer-based program to better suit the needs of children with asthma and their parents.

### Contributions of the Study

This study provides an innovative method for delivering effective asthma education in clinical practice. This may help reduce communication barriers when delivering asthma self-management and promoting medication adherence. Given the cost and resources, this program was proposed to enhance asthma self-management skills through an interactive and growth mindset–enhanced learning environment. Developing a growth mindset may contribute to increased self-confidence and problem solving to face asthma difficulties in daily life among children, thus leading them to be successful managers of their disease in the future.

### Conclusions

A computer-based asthma self-management program composed of an interactive narrative and a serious game was developed in the Indonesian language to overcome the adversities of asthma education in children. The participatory approach, scientific framework, and culture-specific elements led to the successful development of an interactive narrative and serious game at the level of understanding and mindset of children.

## Appendix

Multimedia Appendix 1 Suggestions from the experts and content validity index.

Multimedia Appendix 2 Structure of the storylines and real screenshots.

Multimedia Appendix 3 Elements of asthma self-management.

## Abbreviations

CVI: content validity index
I-CVI: item-specific content validity index
MIRACLE: Module of Inhaler and Asthma Triggers for Children
NEMD: Norma Engaging Multimedia Design

## References

[1] Knox BL, Luyet FM, Esernio-Jenssen D (2019). Medical neglect as a contributor to poorly controlled asthma in childhood. J Child Adol Trauma.

[2] Widayati A, Virginia DM, Setiawan CH, Fenty F, Donowati MW, Christasani PD, Hartayu TS, Suhadi R, Saini B, Armour C (2018). Pharmacists' views on the development of asthma pharmaceutical care model in Indonesia: a needs analysis study. Res Social Adm Pharm.

[3] Farzandipour M, Nabovati E, Sharif R, Arani MH, Anvari S (2017). Patient self-management of asthma using mobile health applications: a systematic review of the functionalities and effects. Appl Clin Inform.

[4] Bellin MH, Newsome A, Lewis-Land C, Kub J, Mudd SS, Margolis R, Butz AM (2018). Improving care of inner-city children with poorly controlled asthma: what mothers want you to know. J Pediat Health Care.

[5] McPherson AC (2006). A randomized, controlled trial of an interactive educational computer package for children with asthma. Pediatrics.

[6] Dweck CS, Yeager DS (2019). Mindsets: a view from two eras. Perspect Psychol Sci.

[7] Pradel FG, Hartzema AG, Bush PJ (2001). Asthma self-management: the perspective of children. Patient Edu Counsel.

[8] Donohoe C, Topping K, Hannah E (2012). The impact of an online intervention (Brainology) on the mindset and resiliency of secondary school pupils: a preliminary mixed methods study. Edu Psychol.

[9] Burkhart PV, Rayens MK (2005). Self-concept and health locus of control: factors related to children's adherence to recommended asthma regimen. Pediatr Nurs.

[10] Whiteley L, Mena L, Craker LK, Healy MG, Brown LK (2019). Creating a theoretically grounded gaming app to increase adherence to pre-exposure prophylaxis: lessons from the development of the viral combat mobile phone game. JMIR Serious Games.

[11] Kato PM, Cole SW, Bradlyn AS, Pollock BH (2008). A video game improves behavioral outcomes in adolescents and young adults with cancer: a randomized trial. Pediatrics.

[12] Howell KJ (2005). “Quest for the Code”: A study of a computer-based education program for children with asthma. Thesis and Dissertations - Syracuse University.

[13] Bergeron BP (2008). Stud Health Technol Inform.

[14] Verschueren S, Buffel C, Stichele G (2019). Developing theory-driven, evidence-based serious games for health: framework based on research community insights. JMIR Serious Games.

[15] Gerwin RL, Kaliebe K, Daigle M (2018). The interplay between digital media use and development. Child Adolesc Psychiatr Clin N Am.

[16] Charlier N, Zupancic N, Fieuws S, Denhaerynck K, Zaman B, Moons P (2016). Serious games for improving knowledge and self-management in young people with chronic conditions: a systematic review and meta-analysis. J Am Med Inform Assoc.

[17] Wyatt TH, Li X, Huang Y, Farmer R, Reed D, Burkhart PV (2013). Developing an interactive story for children with asthma. Nurs Clin North Am.

[18] Mitchell SJ, Bilderback AL, Okelo SO (2016). Racial disparities in asthma morbidity among pediatric patients seeking asthma specialist care. Acad Pediat.

[19] McCallum GB, Morris PS, Brown N, Chang AB (2017). Culture-specific programs for children and adults from minority groups who have asthma. Cochrane Data Syst Rev.

[20] Al Sayah F, Majumdar SR, Williams B, Robertson S, Johnson JA (2012). Health literacy and health outcomes in diabetes: a systematic review. J Gen Intern Med.

[21] Murray L, Elmer S, Elkhair J (2018). Perceived barriers to managing medications and solutions to barriers suggested by bhutanese former refugees and service providers. J Transcult Nurs.

[22] McMillan SS, King M, Tully MP (2016). How to use the nominal group and Delphi techniques. Int J Clin Pharm.

[23] Hsu C, Sandford B (2007). The Delphi Technique: Making sense of consensus. Pract Assess Res Eval.

[24] Abrar EA, Yusuf S, Sjattar EL, Rachmawaty R (2020). Development and evaluation educational videos of diabetic foot care in traditional languages to enhance knowledge of patients diagnosed with diabetes and risk for diabetic foot ulcers. Primary Care Diabetes.

[25] Almanasreh E, Moles R, Chen TF (2019). Evaluation of methods used for estimating content validity. Res Soc Administ Pharm.

[26] Polit DF, Beck CT, Owen SV (2007). Is the CVI an acceptable indicator of content validity? Appraisal and recommendations. Res Nurs Health.

[27] Noenoeng RC, Kartasasmita C, Supriyatno B, Darmawan BS (2017). Pedoman Nasional Asma Anak.

[28] Arnab S, Lim T, Carvalho M, Bellotti F, de FS, Louchart S (2015). Mapping learning and game mechanics for serious games analysis. Br J Edu Technol.

[29] Miracle game edukasi asma. APKPure.

[30] Aronson BD, Janke KK, Traynor AP (2012). Investigating Student Pharmacist Perceptions of Professional Engagement Using a Modified Delphi Process. AJPE.

[31] Louis-Philippe BH, Levy M, Cruz A, Decker R Global initiative for asthma: Asthma management and prevention for adults and children older than 5 years 2020. Global Initiative for Asthma.

[32] Buffel C, van Aalst J, Bangels A, Toelen J, Allegaert K, Verschueren S, Stichele G (2019). A web-based serious game for health to reduce perioperative anxiety and pain in children (CliniPup): pilot randomized controlled trial. JMIR Serious Games.

[33] Suppan M, Gartner B, Golay E, Stuby L, White M, Cottet P, Abbas M, Iten A, Harbarth S, Suppan L (2020). Teaching adequate prehospital use of personal protective equipment during the COVID-19 pandemic: development of a gamified e-learning module. JMIR Serious Games.

[34] Huss K, Winkelstein M, Nanda J, Naumann P, Sloand E, Huss R (2003). Computer game for inner-city children does not improve asthma outcomes. J Pediat Health Care.

[35] Kaufman D, Sauvé L, Renaud L (2011). Enhancing learning through an online secondary school educational game. J Edu Comput Res.

[36] Trollvik A, Ringsberg KC, Silén C (2013). Children's experiences of a participation approach to asthma education. J Clin Nurs.

[37] Mickel CF, Shanovich KK, Evans MD, Jackson DJ (2016). Evaluation of a school-based asthma education protocol. J School Nurs.

[38] Lee T, Sheu S, Chang H, Hung Y, Tseng L, Chou S, Liang T, Liu H, Lu H, Chen M, Liu Y, Tsai C, Sun J (2019). Developing a web-based comic for newly diagnosed women with breast cancer: an action research approach. J Med Internet Res.

[39] Drummond D, Monnier D, Tesnière A, Hadchouel A (2017). A systematic review of serious games in asthma education. Pediatr Allergy Immunol.

[40] Yawn B, Algatt-Bergstrom P, Yawn R, Wollan P, Greco M, Gleason M (2000). An in-school cd-rom asthma education program. J Sch Health.

[41] Shames RS, Sharek P, Mayer M, Robinson TN, Hoyte EG, Gonzalez-Hensley F, Bergman DA, Umetsu DT (2004). Effectiveness of a multicomponent self-management program in at-risk, school-aged children with asthma. Ann Allergy Asthma Immunol.

[42] Haimovitz K, Dweck CS (2017). The origins of children's growth and fixed mindsets: new research and a new proposal. Child Dev.

[43] AlMarshedi A, Wills G, Ranchhod A (2017). Guidelines for the gamification of self-management of chronic illnesses: multimethod study. JMIR Serious Games.

[44] Cutumisu M, Ghoman SK, Lu C, Patel SD, Garcia-Hidalgo C, Fray C, Brown MR, Greiner R, Schmölzer GM (2020). Health care providers’ performance, mindset, and attitudes toward a neonatal resuscitation computer-based simulator: empirical study. JMIR Serious Games.

[45] Poureslami I, Nimmon L, Doyle-Waters M, Rootman I, Schulzer M, Kuramoto L, FitzGerald JM (2012). Effectiveness of educational interventions on asthma self-management in Punjabi and Chinese asthma patients: a randomized controlled trial. J Asthma.

[46] Setiawan C, Widayati A, Virginia DM, Armour C, Saini B (2019). The role of pharmacists in the pharmaceutical care of asthma patients in Yogyakarta, Indonesia: the patients’ views. J Asthma.

[47] Juniper EF, Gruffydd-Jones K, Ward S, Svensson K (2010). Asthma Control Questionnaire in children: validation, measurement properties, interpretation. Eur Resp J.

[48] Indonesia KK (2017). Petunjuk teknis standar pelayanan kefarmasian di apotek. Direktorat Jenderal Kefarmasian dan Alat Kesehatan (Farmalkes).

