# Supplementary material for: A Computer-Based Interactive Narrative and a Serious Game for Children With Asthma: Development and Content Validity Analysis
Source: J Med Internet Res. 2021 Sep 13;23(9):e28796. doi: 10.2196/28796 (PMC8477291; doi:10.2196/28796)
Supplement: Multimedia Appendix 1 [file jmir_v23i9e28796_app1.docx]

**Multimedia Appendix 1. Suggestions from the experts and content validity index.**

**Table Lists**

**Table S1.** Suggestions from the experts in Delphi round 1…………………………… 2

**Table S2.** Evaluation of content validity index in Delphi round 2……………………. 6

**Table S3.** Agreement in Delphi……………………………………………………….. 9

**Delphi Round 1 (*n* = 40 experts)**

1 open question raised, “According to your experience, what are the components of an interactive narrative and serious game that children can understand about asthma?*”.*

**Table S1.** Suggestions from the experts in Delphi round 1.

| No. of expert | Suggestions from experts in Delphi round 1 | |
| --- | --- | --- |
| Suggestions | Elements of asthma self-management | Design of the interactive narrative and serious game |
| 1 | Introduction of asthma triggers, prevention of asthma triggers, administration of asthma medications, self-management, first-aid knowledge. | Cartoons, colorful design and layout, contain plots or narrative with specific purposes. |
| 2 | Asthma attacks and how to prevent asthma attacks. | Cartoons with animation and an entertaining game. |
| 3 | Basic knowledge of asthma, asthma triggers, management of treatment (pharmacological and non-pharmacological, including asthma education), increase self-confidence. | Interactive layout, easy to understand, use mother language (mother tongue), colorful design. |
| 4 | Signs and symptom of asthma, first-aid asthma attack exposure. | Cartoons, colorful design, challenges. |
| 5 | Asthma triggers, type of asthma triggers, and how to prevent asthma triggers. | Eye-catching design, the layout and design should represent children’s environments. |
| 6 | What is asthma, pathophysiology of asthma, normal airway, what should children do when asthma attack occurs, and dos and don’ts for asthma children in daily life. | Colorful design for narrative and game. |
| 7 | Signs and symptoms of asthma, self-management, inhaler techniques (how to use, clean, and store the inhaler, and expiration date). | The narrative and game should be easy to understand and play, simple, and have a colorful design. |
| 8 | What is asthma, what are asthma triggers, signs and symptoms of asthma, how to resolve an asthma attack, how to prevent asthma attacks, how to control asthma, dos and don’ts for asthma children, proper inhaler technique. | Attractive color design, provide examples and pictures of dosage forms in the narrative and game. |
| 9 | Asthma definition, normal airways, asthma etiology, asthma medications, first-aid knowledge, asthma is not a contagious disease. | Narrative should contain less text and provide more-colorful design. Game should engage children and provide scenarios. |
| 10 | Asthma definition, signs and symptoms of asthma, asthma treatment and management. | Narrative contains illustrations or comics and less text. |
| 11 | Asthma definition, signs and symptoms of asthma, treatment and medication, prevention of asthma attacks. | Less text in the narrative and more-attractive performance or animation in the game. |
| 12 | Asthma definition, treatment and medications, prevention of asthma attacks. | Simple and easy to understand, texts should not be ambiguous, colorful design. |
| 13 | Asthma definition, signs and symptoms of asthma, how to prevent asthma attacks. | Cartoons of an asthma attack in children, provide cartoons to show procedures of proper inhaler techniques. |
| 14 | How to prevent asthma attacks, non-pharmacological treatment for asthma to prevent asthma attacks or asthma exacerbation, how to control asthma, how to use asthma medications, especially for inhaler, nebulizer, etc, the importance of medication adherence when taking asthma medications. | Cartoons or 3D animation with positive characters (happy face, smile, motivate children to read and play). |
| 15 | Asthma definition, signs and symptoms of asthma, when asthma attack occurs, how to solve asthma attacks, asthma medications and their dosage forms. | The narrative and the game should contain storyline or scenario to engage children to read and play. |
| 16 | Signs and symptoms of asthma, how to prevent asthma attacks, what should children do when an asthma attack occurs. | The narrative should be easy to read and the game should contain animation and music. |
| 17 | Definition of asthma in children, asthma triggers, asthma is not a contagious disease, children with asthma can live normally, how to prevent asthma triggers, what should children do when an asthma attack occurs, what kind of sports that children with asthma can do, what kind of medications that children should bring every time they go outside. | The narrative and game should contain cartoons with children, family (support system), asthma triggers, and their healthy daily lifestyle. |
| 18 | How to prevent asthma triggers, recognize asthma attacks, always prepare asthma medications (instruction, how to use medication effectively), how to store medications, and know the most appropriate time to use inhaler and the expiration date. | Cartoons or illustrations to show the content of asthma, contain storyline or sessions (levels or missions), easy to follow or understand. |
| 19 | Normal airways, asthma definition, pathophysiology of asthma, dos and don’ts for children with asthma, what should children do when an asthma attack occurs, how to properly use an inhaler. | Colorful with animations or visualizations of children or animals (elementary school). |
| 20 | Process from normal airways to an asthma attack, what children should do when an asthma attack occurs, asthma medications, how to use asthma medications at their level of understanding. | Colorful design with storyline or comics with less text. |
| 21 | Asthma definition, risk factors, asthma triggers, tips and tricks in daily life for asthmatic children, medication adherence. | Colorful design in the narrative and a game in an urban living environment. |
| 22 | Asthma etiology, asthma triggers, how to prevent asthma triggers, how to resolve an asthma attack. | Colorful design, contain storylines and purpose. |
| 23 | At a glance of asthma definition, signs and symptoms of asthma, procedures to resolve an asthma attack, prevention of asthma triggers, asthma control in daily life. | Colorful design, contain storyline, provide cartoons or characters, or artificial intelligent features (if possible). |
| 24 | Asthma definition, dos and don’ts for children with asthma, asthma attacks, medications and treatments at the level of children’s understanding. | Educative storyline with purpose and easy to understand for children (elementary school and teens). |
| 25 | Asthma definition, asthma triggers, how to prevent asthma triggers, first-aid action when an asthma attack occurs at home. | Colorful design and simple layout with storyline or missions to complete. |
| 26 | Asthma definition, signs and symptoms of asthma, asthma medications and their dosage forms (inhaler techniques). | Colorful design, appropriate for children and adolescents. |
| 27 | Asthma definition, asthma etiology, pathophysiology of asthma. | Colorful design, eye-catching design or layout, contain storyline, use mother language or national language that is easy to understand. |
| 28 | Asthma definition, signs and symptoms of asthma, asthma triggers, asthma medications and treatment, proper inhaler techniques, when to take asthma medications, how to store asthma medications. | Colorful design, eye-catching layout, cartoons especially for procedures of taking metered-dose inhaler, and less text. |
| 29 | Asthma definition, asthma triggers, signs and symptoms of asthma, how to avoid asthma triggers, how to resolve an asthma attack. | 2D or 3D comics that contain a storyline, easy to understand for children and adolescents. |
| 30 | Asthma definition, asthma etiology, asthma medications and treatment, prevention of asthma attacks, risk factors, dos and don’ts for children with asthma, how to control asthma. | Comics that contain a storyline, allow children to make their own decisions, colorful design. |
| 31 | Asthma triggers, first-aid knowledge, how to use asthma medications. | Colorful design, fun and positive characters, provide 2D-3D animation. |
| 32 | Asthma definition, asthma triggers, how to prevent asthma triggers, parents’ role in managing and identifying asthma in children, self-management and confidence. | Colorful design with game flow, provide fun, positive, and expressive characters, suitable for children and adolescents. |
| 33 | Signs and symptoms of asthma, what should children do when an asthma attack occurs. | Colorful design, less text, use national language. |
| 34 | Asthma triggers, how to handle an asthma attack, signs and symptoms of asthma attacks, asthma medications. | Colorful design, contain storyline or missions to complete (challenges). |
| 35 | Pathophysiology of asthma in general that children can easily understand, identification of asthma triggers, how to prevent asthma triggers, how to control asthma triggers in the environment to prevent asthma attacks, healthy daily lifestyles, what should children do when an asthma attack occurs at home, how to take asthma medications, emergency conditions, when is the appropriate time to go to the hospital or emergency department. | Colorful design, provide 2D-3D animation, can be applied for children (5-12 years old) and adolescents (12-18 years old). |
| 36 | Asthma definition, signs and symptoms of asthma, asthma triggers, how to prevent asthma attacks, daily life for asthmatic children, and what should children do when an asthma attack occurs. | Cartoons with positive characters that represent children’s environments, represent children’s activities in daily life, colorful design. |
| 37 | Asthma definition, asthma etiology, signs and symptoms of asthma, medications, non-pharmacological treatments. | Interactive narrative with storyline, colorful design, and layout, containing missions to complete or goals (achievements). |
| 38 | Pathophysiology of asthma, risk factors, asthma triggers, asthma medications, adverse drug reactions, management of using medications. | Colorful design, containing animations or storyline, battle with asthma triggers, containing motivation for children. |
| 39 | Asthma definition, pathophysiology of asthma, signs and symptoms of asthma, asthma triggers and their prevention, warning signs of asthma attacks, when the children should go to the doctor and hospital, emergency conditions, first-aid knowledge. | Cartoons with animation, colorful designs, containing simple game, like puzzles, platforms, or battles, and a narrative containing storylines. |
| 40 | Asthma definition, asthma triggers, pathophysiology of asthma, prevention of asthma triggers, type of asthma medications, administration of asthma medications, and their dosage forms (inhaler, nebulizer, or syrup). | Colorful design and layout, cartoon with 2D-3D animation, appropriate for children and adolescents, interactive story (appropriate for story telling) |

**Delphi round 2 (*n* = 38 experts)**

Delphi round 2 generated 38 item statements from Delphi round 1, consisting of two parts, including:

- Part 1: elements of asthma self-management
- Part 2: design of the interactive narratives and serious game.

Thirty-eight item statements were constructed with four-point Likert scale and was deployed to 38 experts.

**Table S2.** Evaluation of content validity index in Delphi round 2

| **#** | **Item statement** | **Level of consensus (*N*=38)** | | | | |
| --- | --- | --- | --- | --- | --- | --- |
|  |  | **Agreement** | **Disagreement** | **Agreement (%)** | **Disagreement (%)** | **Item CVI ^a^** |
| **Part 1: Elements of asthma self-management** | | | | | | |
| 1 | Provide information about normal or healthy airways (lungs). | 38 | 0 | 100 | 0 | 1 |
| 2 | Display information about the condition of the lungs during an asthma attack. | 38 | 0 | 100 | 0 | 1 |
| 3 | Provide information about the definition of asthma. | 38 | 0 | 100 | 0 | 1 |
| 4 | Provide information that asthma can appear anytime and anywhere. | 38 | 0 | 100 | 0 | 1 |
| 5 | Provide information about asthma triggers. | 38 | 0 | 100 | 0 | 1 |
| 6 | Explain how to avoid things that can trigger asthma attacks. | 38 | 0 | 100 | 0 | 1 |
| 7 | Explain the need to recognize asthma triggers themselves and avoid them. | 38 | 0 | 100 | 0 | 1 |
| 8 | Explain the symptoms felt during an asthma attack. | 38 | 0 | 100 | 0 | 1 |
| 9 | Explain the need to recognize and monitor signs of asthma. | 38 | 0 | 100 | 0 | 1 |
| 10 | Explain to seek help immediately when an asthma attack occurs. | 38 | 0 | 100 | 0 | 1 |
| 11 | Explain what to do when an asthma attack occurs. | 37 | 1 | 97.4 | 2.6 | 0.97 |
| 12 | Explain uncontrolled symptoms and signs of asthma. | 37 | 1 | 97.4 | 2.6 | 0.97 |
| 13 | Explain the importance of adherence to medications. | 36 | 2 | 94.7 | 5.3 | 0.97 |
| 14 | Explain about asthma treatment in general, for example drug controller and drug reliever. | 35 | 3 | 92.1 | 7.9 | 0.92 |
| 15 | Explain the indication of inhalers; the purpose of inhalers. | 37 | 1 | 97.4 | 2.6 | 0.97 |
| 16* | Explain the types of inhaler available that can be found in Indonesia | 29 | 9 | 76.3 | 23.7 | 0.76 |
| 17 | Explain how to use an inhaler. | 38 | 0 | 100 | 0 | 1 |
| 18 | Explain how to properly clean the inhaler. | 34 | 4 | 89.5 | 10.5 | 0.89 |
| 19 | Explain how to correctly store inhalers. | 38 | 0 | 100 | 0 | 1 |
| 20 | Explain that asthma is not a contagious disease. | 35 | 3 | 92.1 | 7.9 | 0.92 |
| 21 | Explain that people with asthma can live normally (asthma can be controlled). | 34 | 4 | 89.5 | 10.5 | 0.89 |
| 22 | Explain that asthma drugs do not cause dependence (addiction). | 35 | 3 | 92.1 | 7.9 | 0.92 |
| 23 | Explain the importance of carrying asthma medication when traveling anywhere. | 38 | 0 | 100 | 0 | 1 |
| 24 | Explain that a written action plan is something that needs to be completed. | 31 | 7 | 81.5 | 18.4 | 0.81 |
| 25 | Explain the need for regularly scheduled doctor visits. | 34 | 4 | 89.5 | 10.5 | 0.89 |
| Part 1 | I-CVI (for 25 items) |  |  |  |  | 23.88 |
| **Part 2: Design of the interactive narratives and serious game** | | | | | | |
| 26 | A scenario of asthma attacks is needed. | 37 | 1 | 97 | 2.6 | 1 |
| 27 | An asthma narrative contains conversation between characters. | 38 | 0 | 100 | 0 | 1 |
| 28 | The narrative and game should use Bahasa Indonesian (the local native language). | 38 | 0 | 100 | 0 | 1 |
| 29 | A video is embedded in the narrative. | 38 | 0 | 100 | 0 | 1 |
| 30 | A text box and colorful design are considered in the narrative. | 38 | 0 | 100 | 0 | 1 |
| 31 | Music with a good beat is needed in the narrative and game. | 38 | 0 | 100 | 0 | 1 |
| 32 | Show the correct steps of using an inhaler with positive characters (smiling, happy, and motivated) and music. | 38 | 0 | 100 | 0 | 1 |
| 33 | Show step-by-step demonstration of using an inhaler by children. | 37 | 1 | 97 | 2.6 | 1 |
| 34 | Narrative and a game use simple asthma terminology that children can understand the meaning (inhalation can be defined as “to take a breath, to inhale”). | 38 | 0 | 100 | 0 | 1 |
| 35 | Duration (time) for reading narrative and playing game are needed (min. 5 minutes, max. 30 minutes). | 36 | 2 | 94.7 | 5.3 | 0.97 |
| 36 | Number of game sessions is needed. | 37 | 1 | 97 | 2.6 | 1 |
| 37 | Font should be easy to read and number of plots are needed. | 37 | 1 | 97 | 2.6 | 1 |
| 38 | Minimal text and colorful design are considered in the game. | 37 | 1 | 97 | 2.6 | 1 |
| Part 2 | I-CVI (for 13 items) |  |  |  |  | 12.97 |
| Total | I-CVI (38 items) |  |  |  |  | 36.85 |
|  | Ave-CVI (38 items) |  |  |  |  | 0.96 |
|  | UA-CVI |  |  |  |  | 0.52 |

**Achieved Consensus (*N* = 38 experts)**

The experts agreed to remove item statement no. 16 due to an I-CVI of <0.80, resulting in total 24 item statements of elements of asthma self-management and 13 item statements of design.

**Table S3.** Agreement in Delphi

| **Item** | **Element** | **I-CVI ^a^** | **Avg-CVI ^b^** | **UA-CVI ^c^** |
| --- | --- | --- | --- | --- |
| 16* | Explain the types of inhaler available that can be found in Indonesia (removed) | 0.76 |  |  |
|  | **Total item statements** |  |  |  |
| Part 1 | 24 item statements | 22.12 | 0.92 | 0.52 |
| Part 2 | 13 item statements | 12.97 | 0.99 | 0.53 |

Definitions:

^a^ Item-CVI = Number of experts rating the item either 3 or 4/total number of experts.

^b^ Ave-CVI = Sum of the I-CVIs (I-CVI1+I-CVI2+I-CVI3+ …….+I-CVIn)/total number of items.

^c^ UA-CVI = Number of items that achieved rating 3 or 4 by all experts/total number of items.

Abbreviations:

I-CVI: content validity index of an item; Ave-CVI: content validity index of the entire instrument, averaging calculation method; UA-CVI: content validity index of the entire instrument, Universal agreement calculation method.
