# Supplementary material for: A Computer-Based Interactive Narrative and a Serious Game for Children With Asthma: Development and Content Validity Analysis
Source: J Med Internet Res. 2021 Sep 13;23(9):e28796. doi: 10.2196/28796 (PMC8477291; doi:10.2196/28796)
Supplement: Multimedia Appendix 2 [file jmir_v23i9e28796_app2.docx]

**Multimedia Appendix 2. Structure of the storylines and the real screenshots.**

**Figure lists.**

**Figure S1.** Structure of the storylines…………………………………………………… 2

**Figure S2.** The serious game appearance on Google Playstore platform………………. 3

**Figure S3.** The screenshots of the interactive narrative configuration menu……………. 4

**Figure S4.** The screenshots of the interactive narrative plots menu……………………… 5

**Figure S5.** The screenshots of the game configuration/customization menu……………. 6

**Figure S6.** The screenshots of asthma education in the game……………………….…... 7

**Figure S7.** The screenshots of the game sessions………………………………………… 8

Structure of the storyline in the interactive narrative is described below. The green box means finishing the plots without asthma attack, the yellow box means actuating asthma medications with some concern, and the red box means the ending of the plot with an asthma attack.

**Figure S1.** Structure of the storylines.

**Figure S2.** The serious game appearance on Google Playstore platform.

**
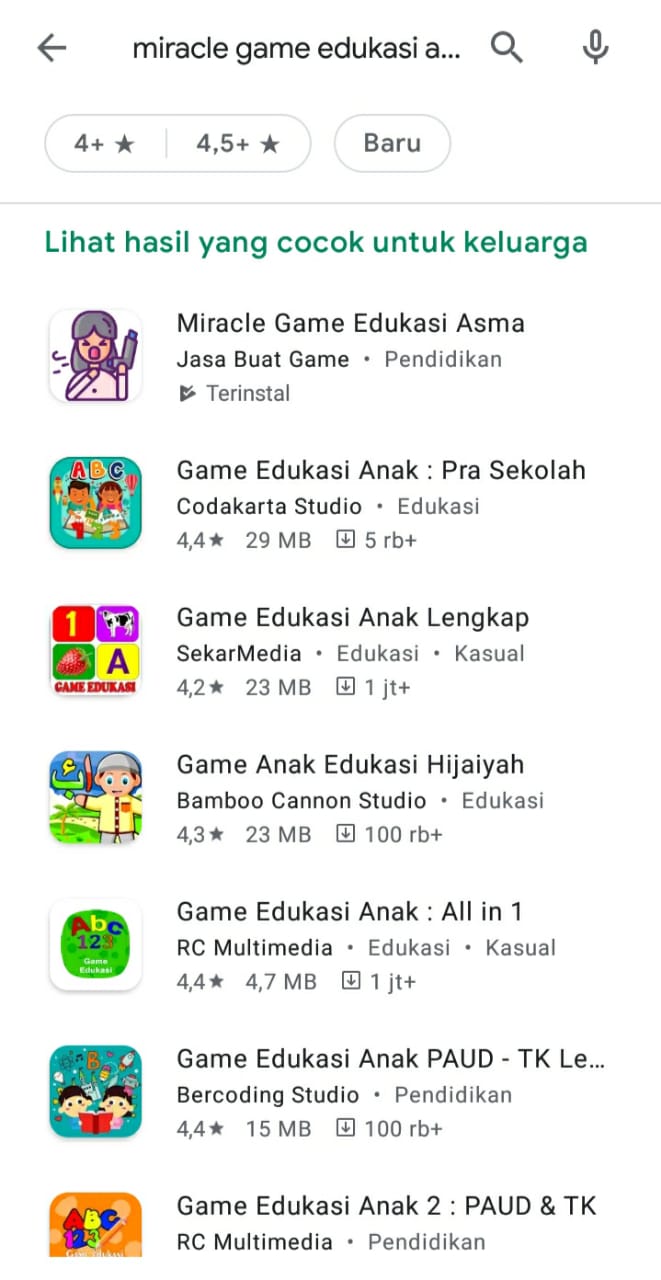

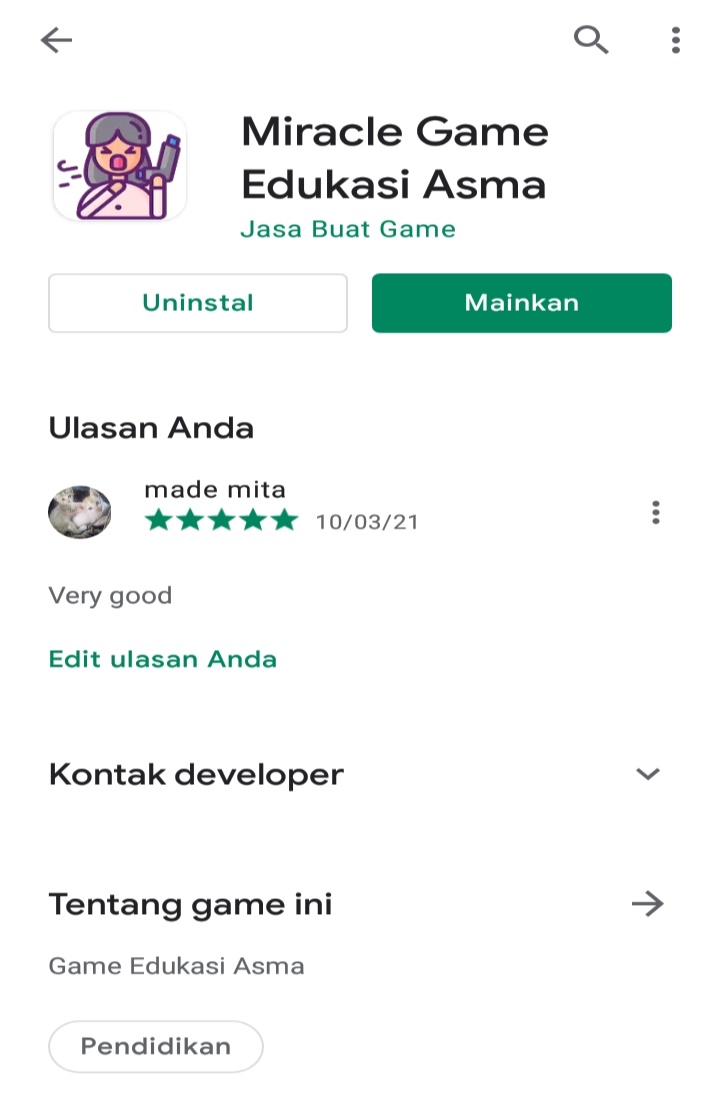
**

URL: <https://play.google.com/store/apps/details?id=com.madearysarasmita.MiracleGameEdukasiAsma>

**Figure S3.** The screenshots of the interactive narrative configuration menu. Introduction and content lists (top left), asthma information (top right), characters (bottom left), and take home messages (bottom right).

| Introduction and content lists.  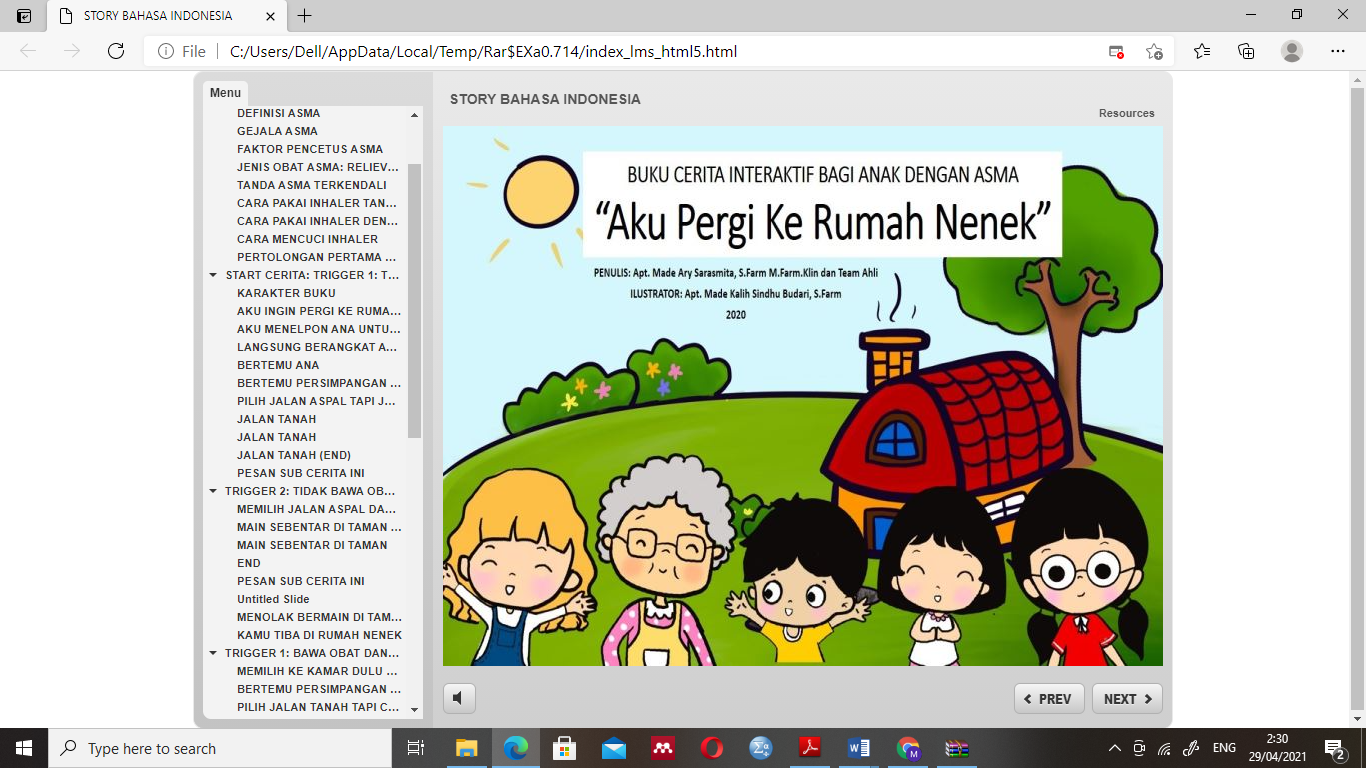 | Asthma information.  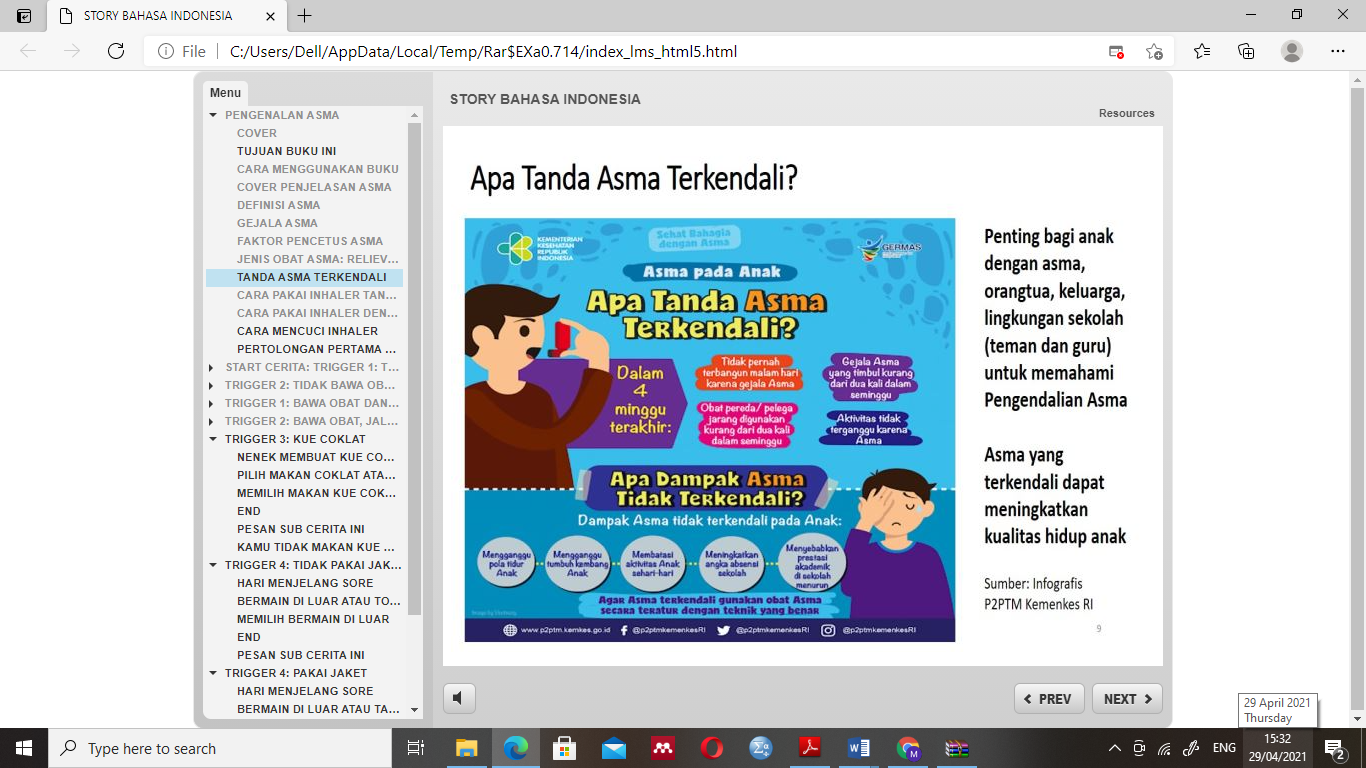 |
| --- | --- |
| The characters.  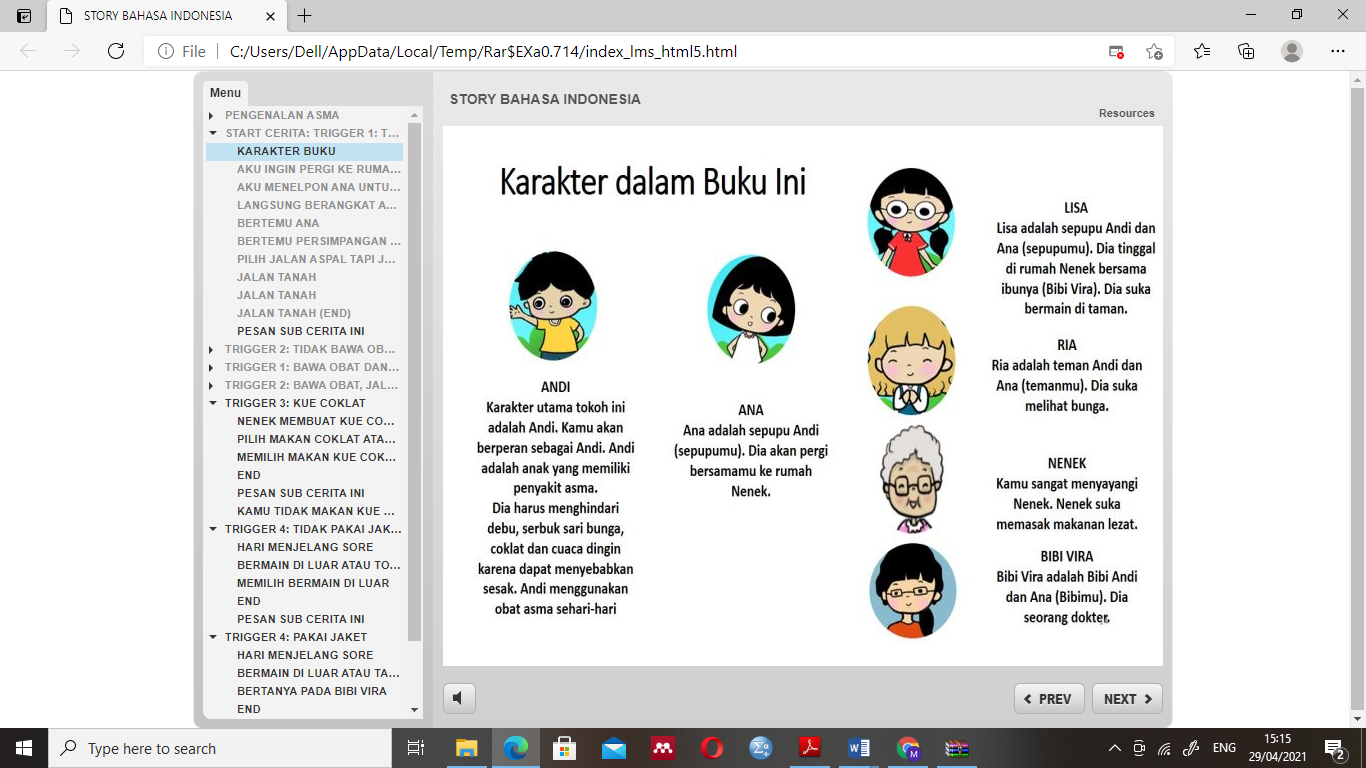 | Take home messages.  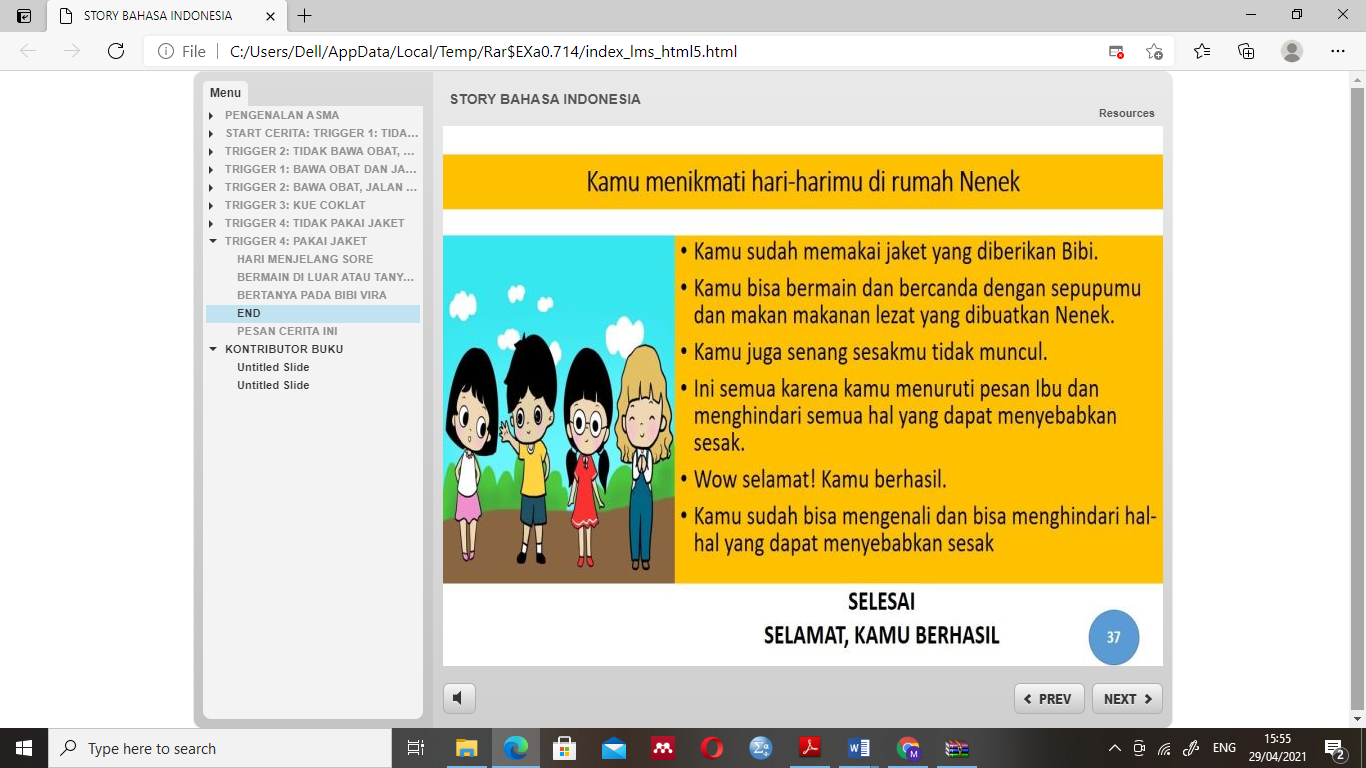 |

**Figure S4.** The screenshots of the interactive narrative plots menu. Plot 1 (top left), plot 2 (top right), plot 3 (bottom left), and plot 4 (bottom right).

| Plot 1: Asthma triggers (the dust and mould).  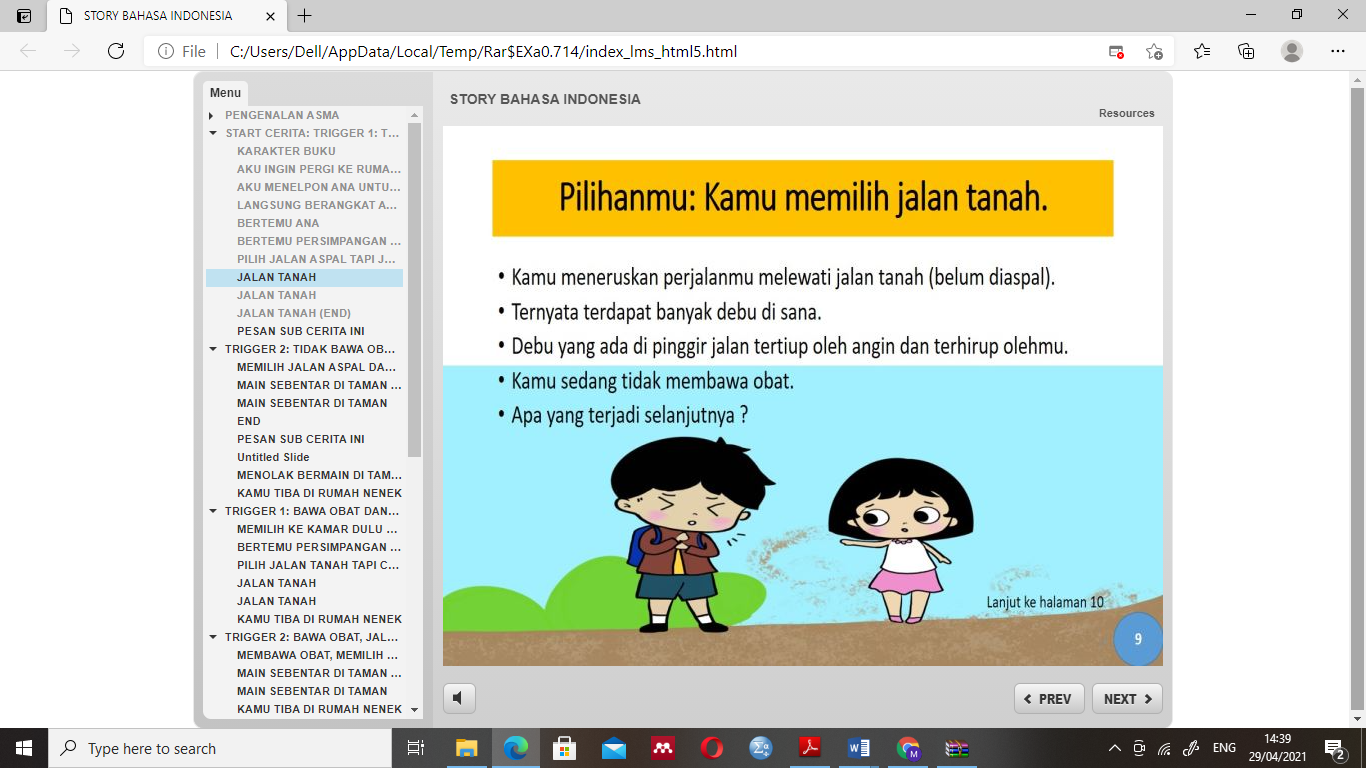 | Plot 2: Asthma triggers (the pollens).  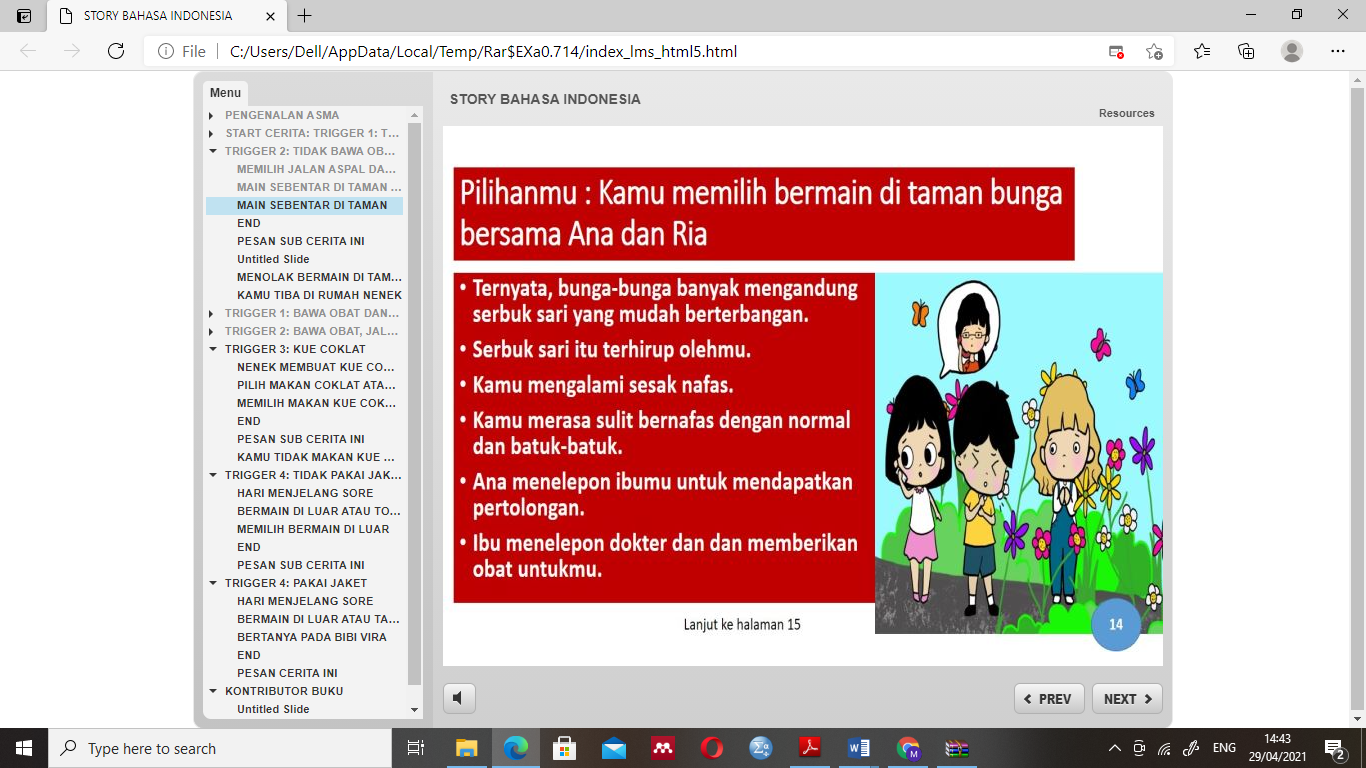 |
| --- | --- |
| Plot 3: Asthma triggers (the food allergies).  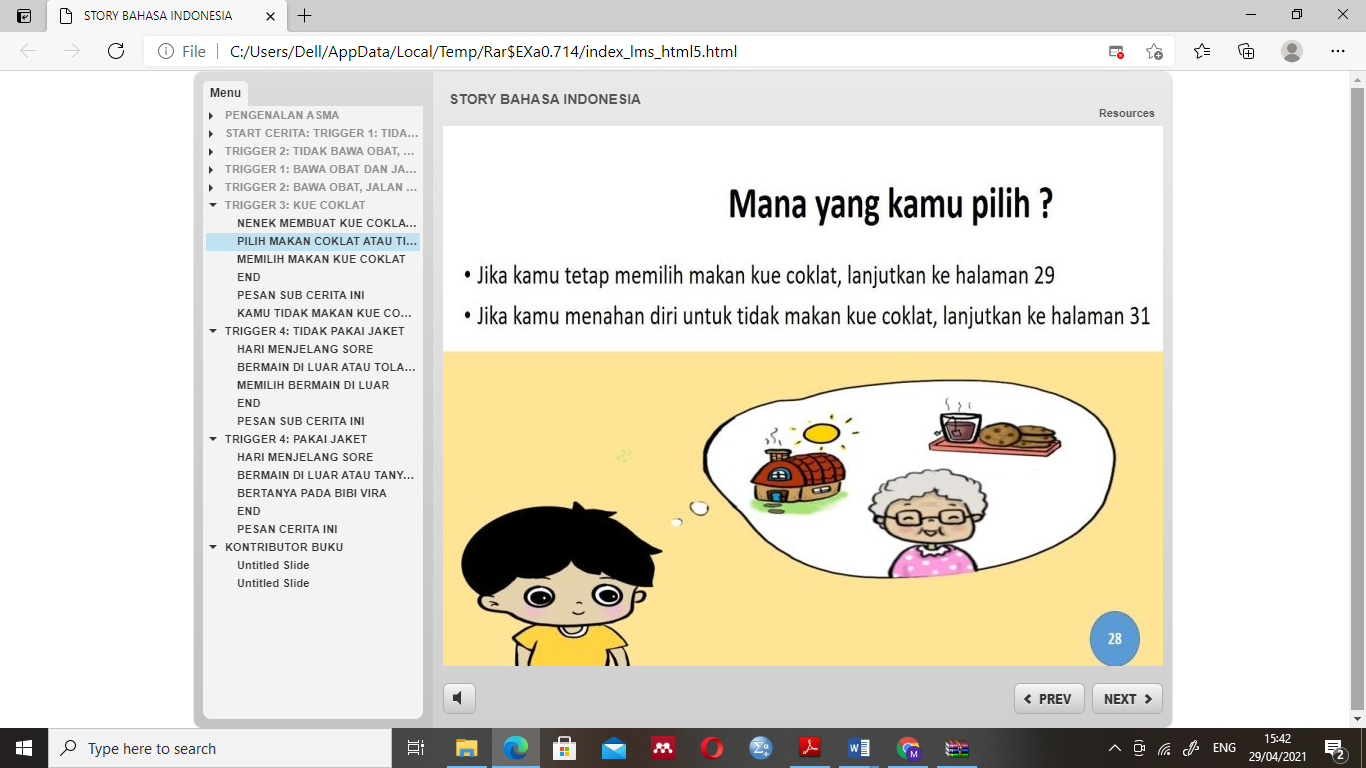 | Plot 4: Asthma triggers (the cold weather).  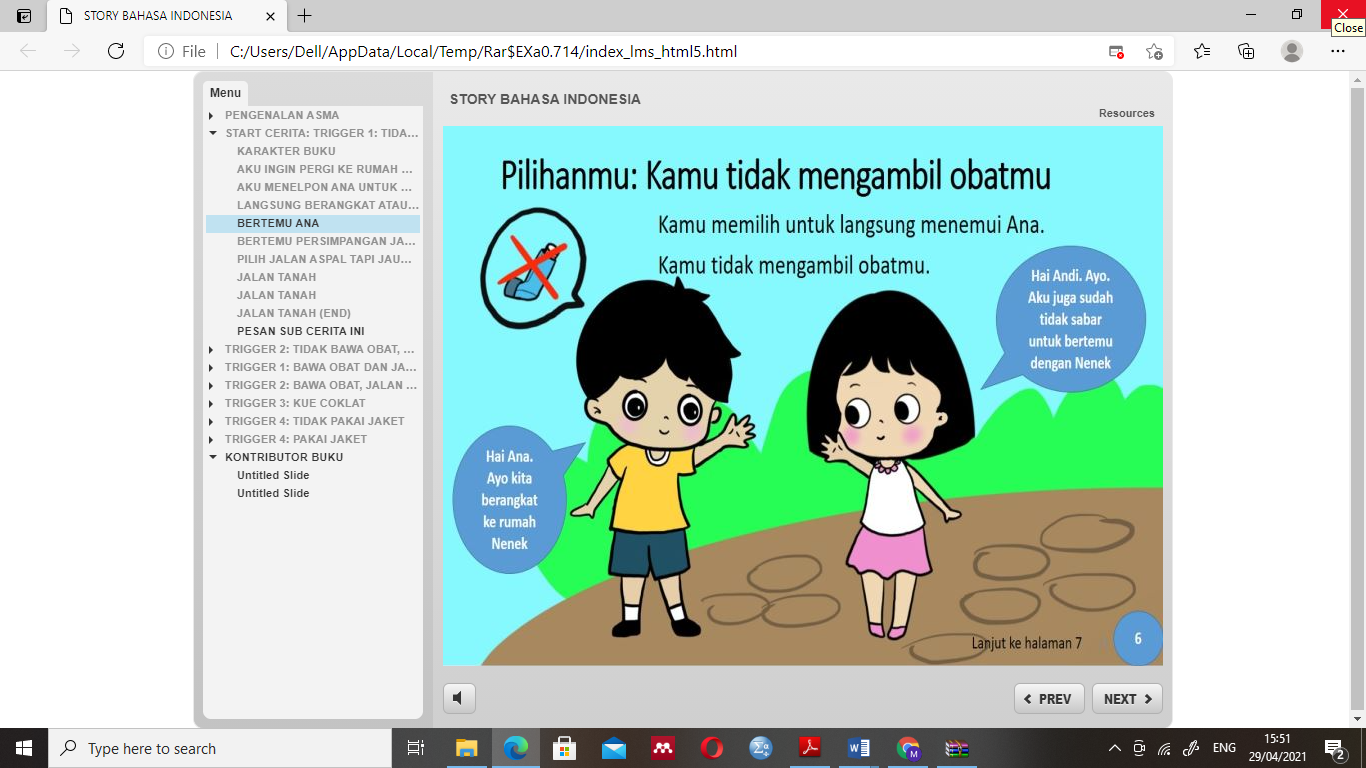 |

**Figure S5.** The screenshots of the game configuration/customization menu. User menu (top left), game story (top right), characters (middle left), game map (middle right), feedback (bottom left), and targeted goals (bottom right).

| 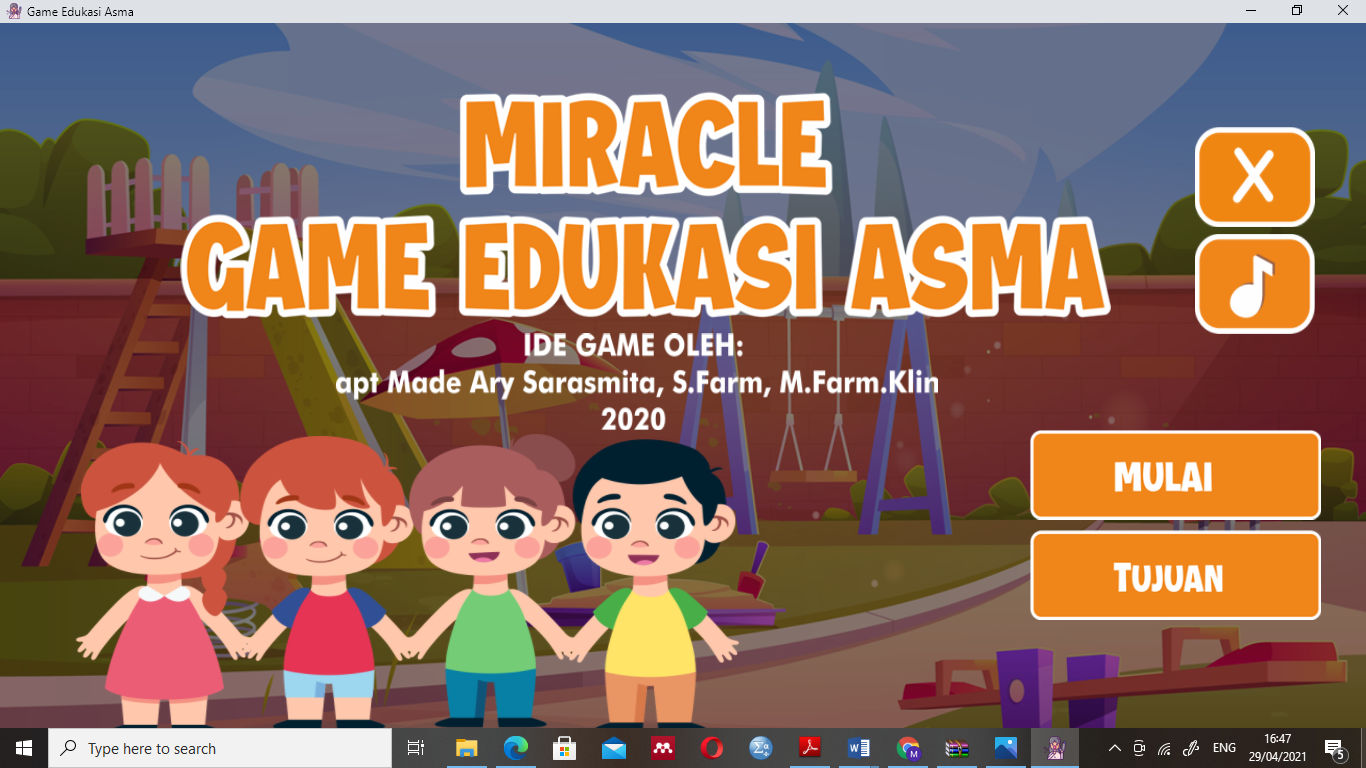 | 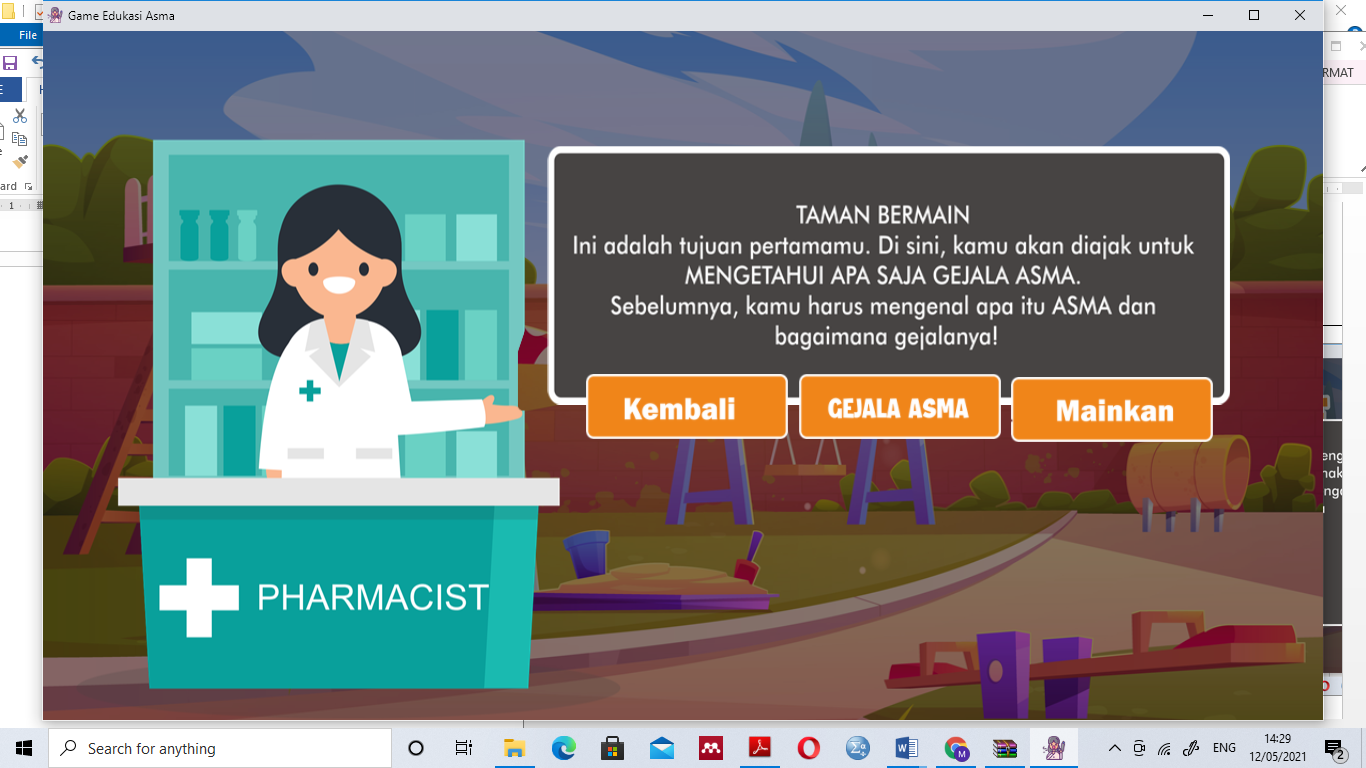 |
| --- | --- |
| 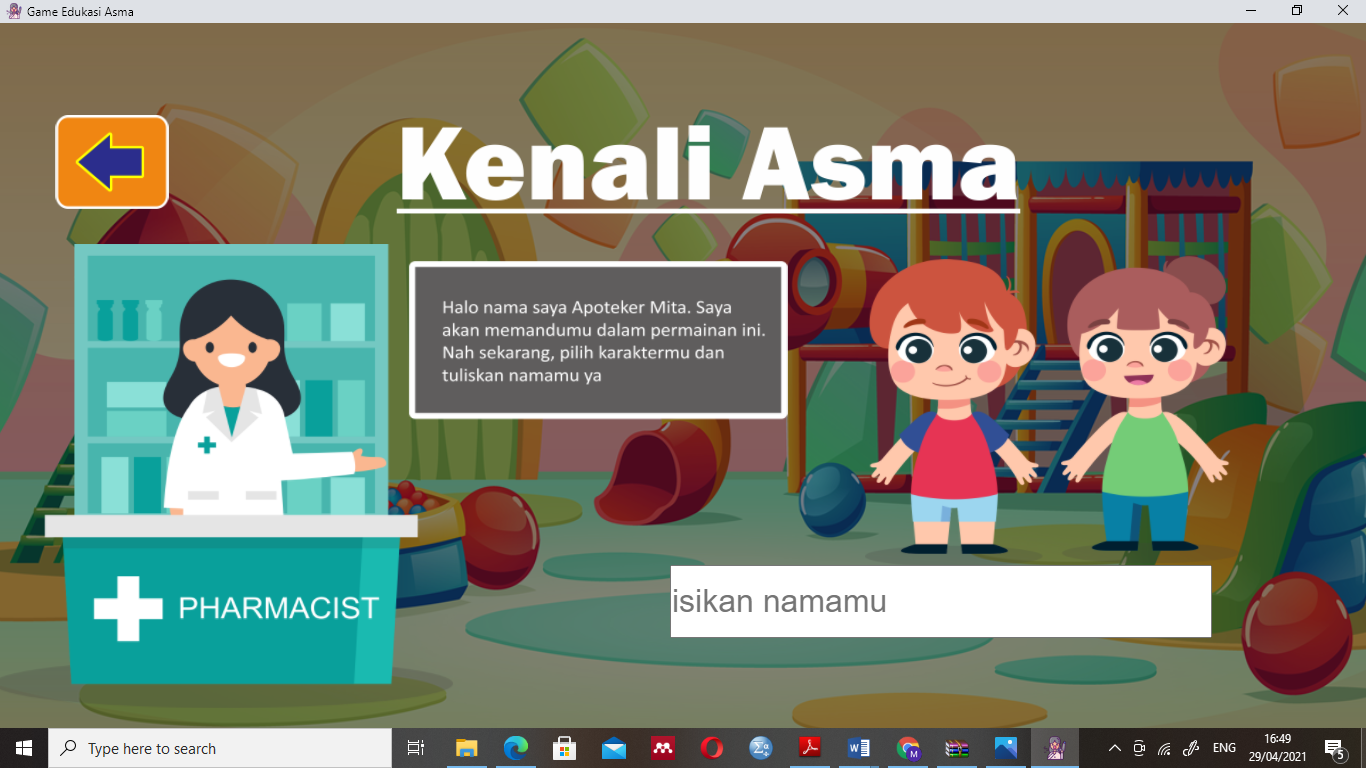 | 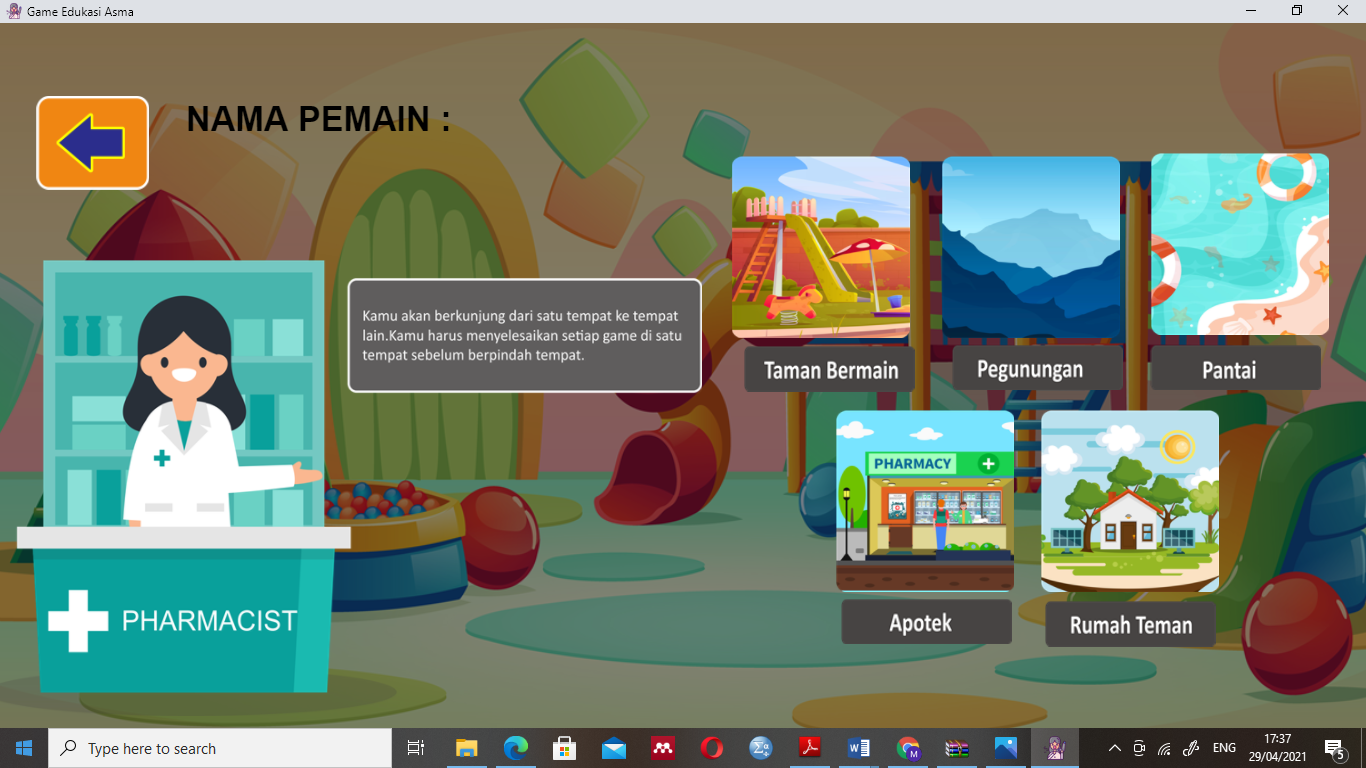 |
| 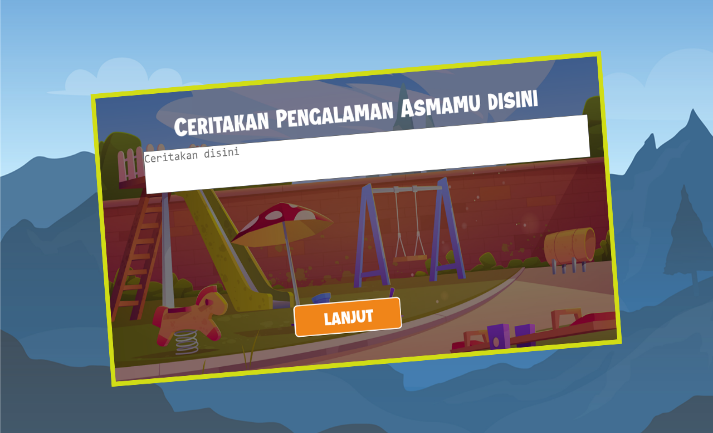 | 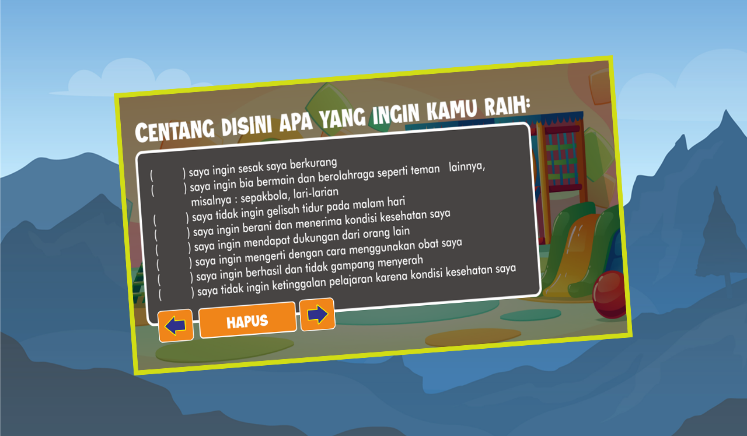 |

**Figure S6.** The screenshots of asthma education in the game. The explanation of asthma triggers prevention (top left), the explanation of asthma signs and symptoms (top right), the explanation of asthma medications (bottom left), and the explanation of controlled asthma (bottom right).

| 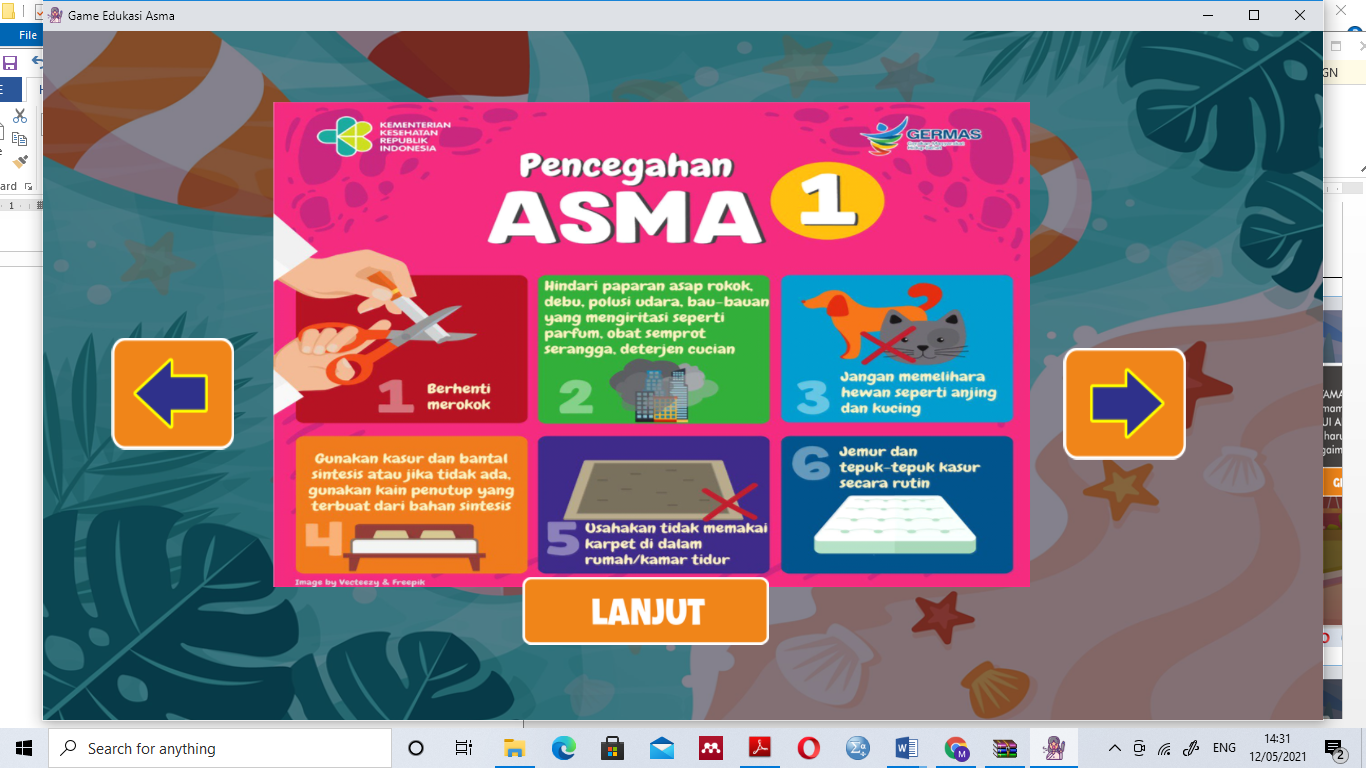 | 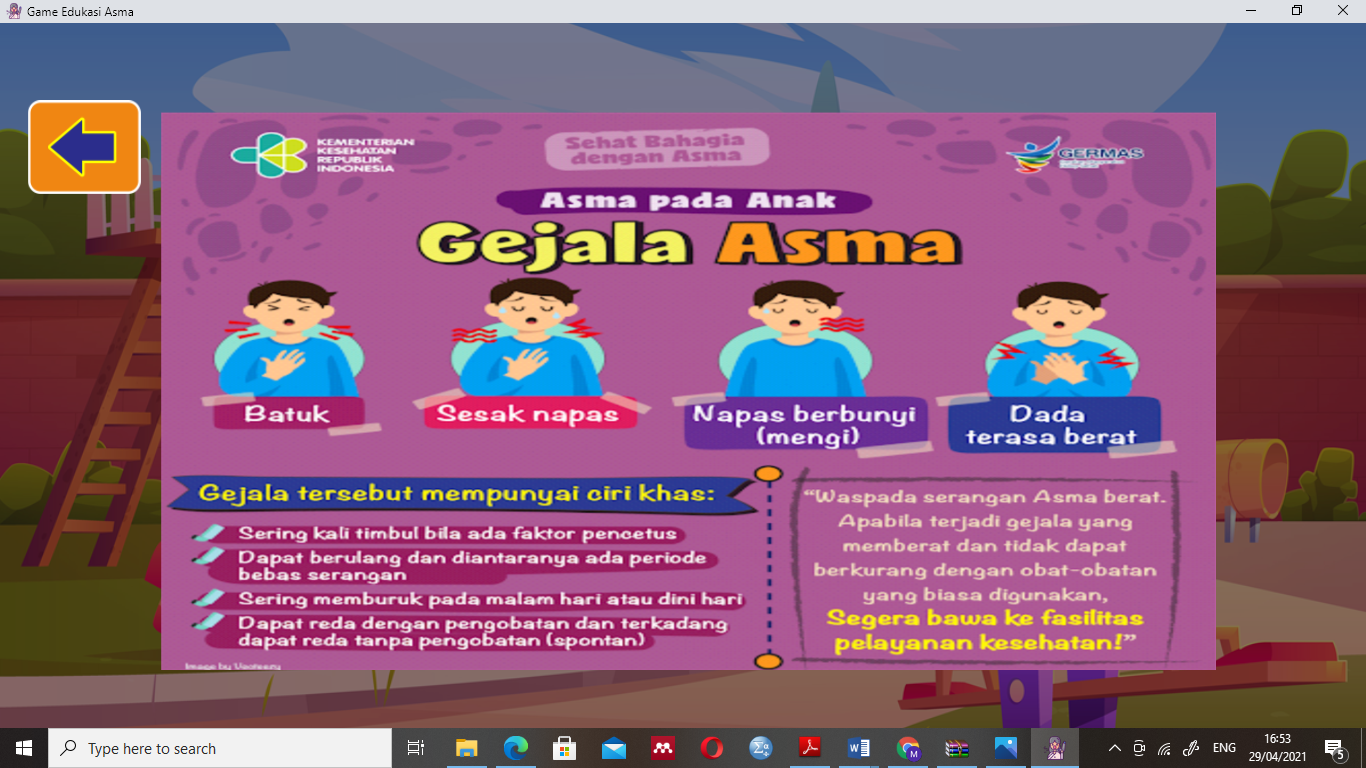 |
| --- | --- |
| 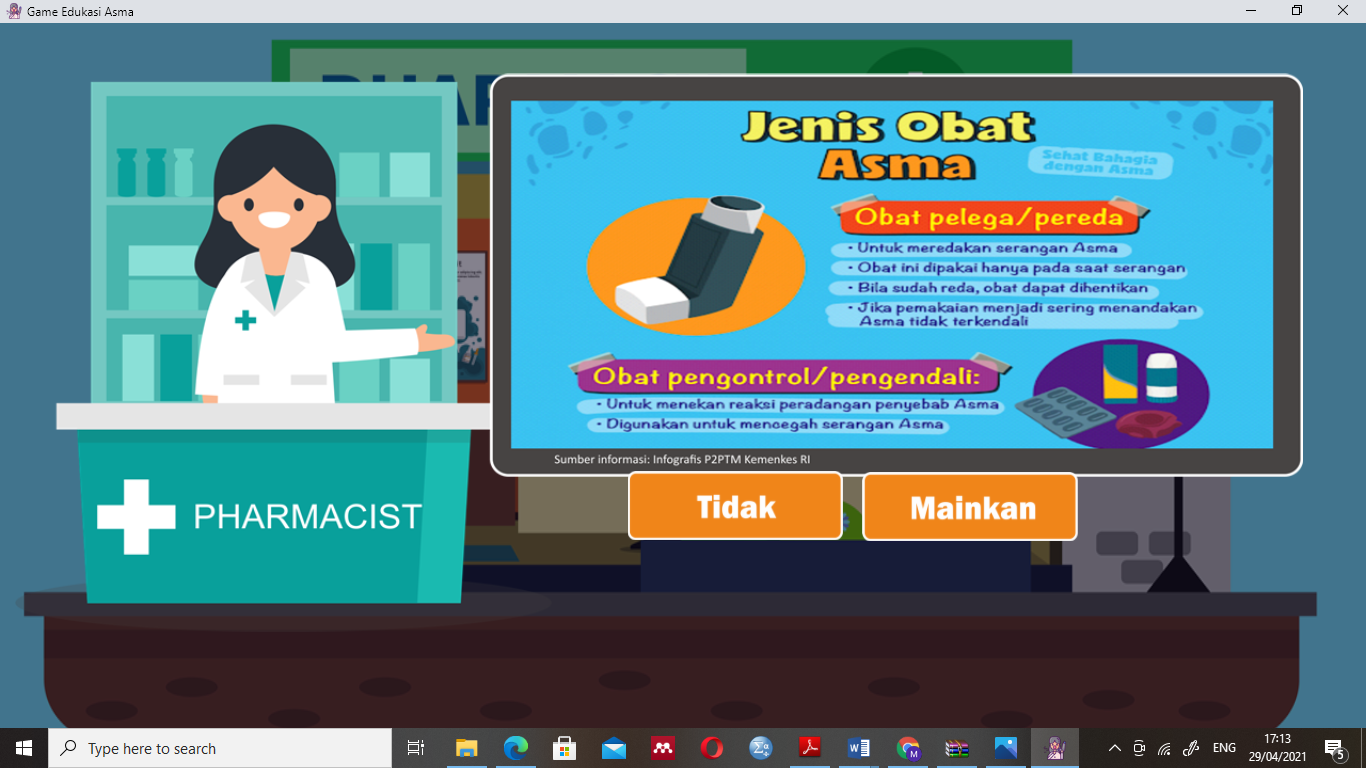 | 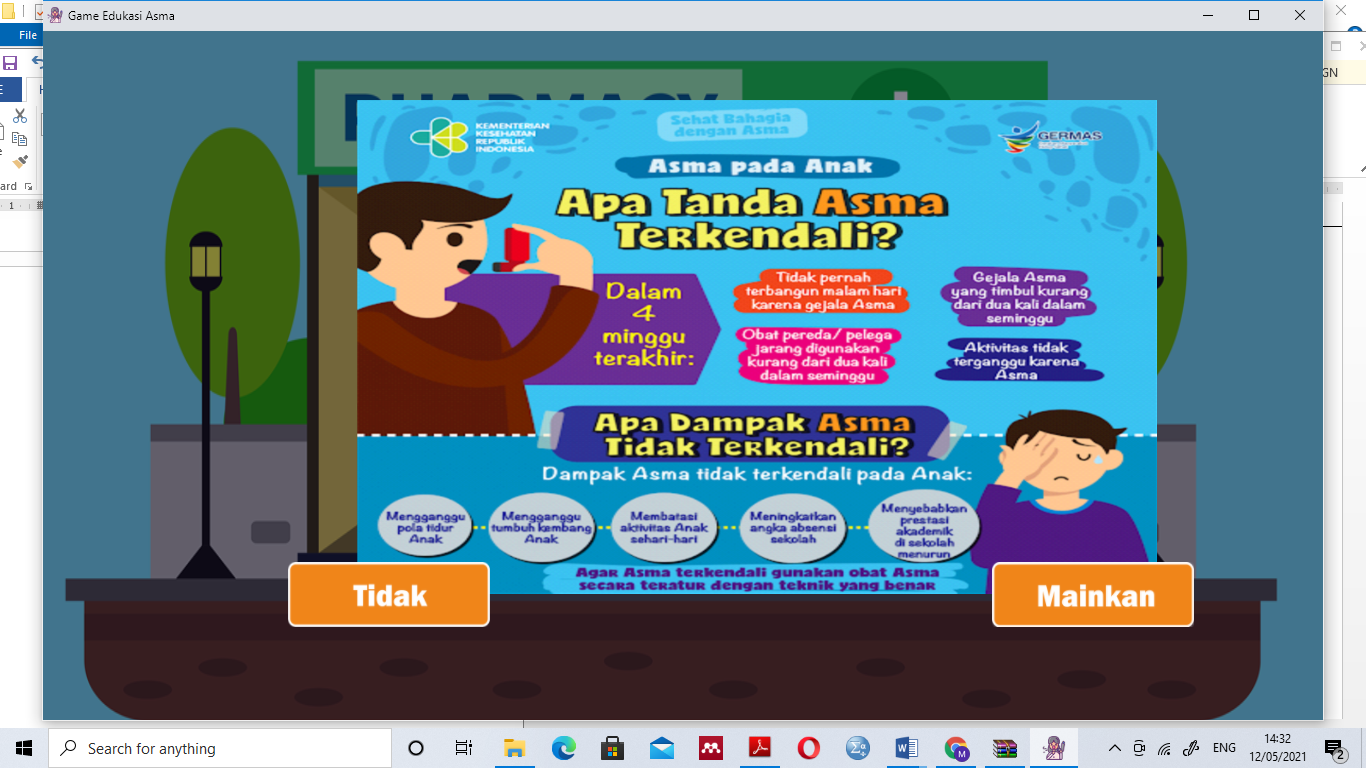 |

**Figure S7.** The screenshots of the game sessions. Game session 1 (top left), game session 2 (top right), game session 3 (middle left), game session 4 (middle right), game session 5 (bottom left), and scoring (bottom right).

| 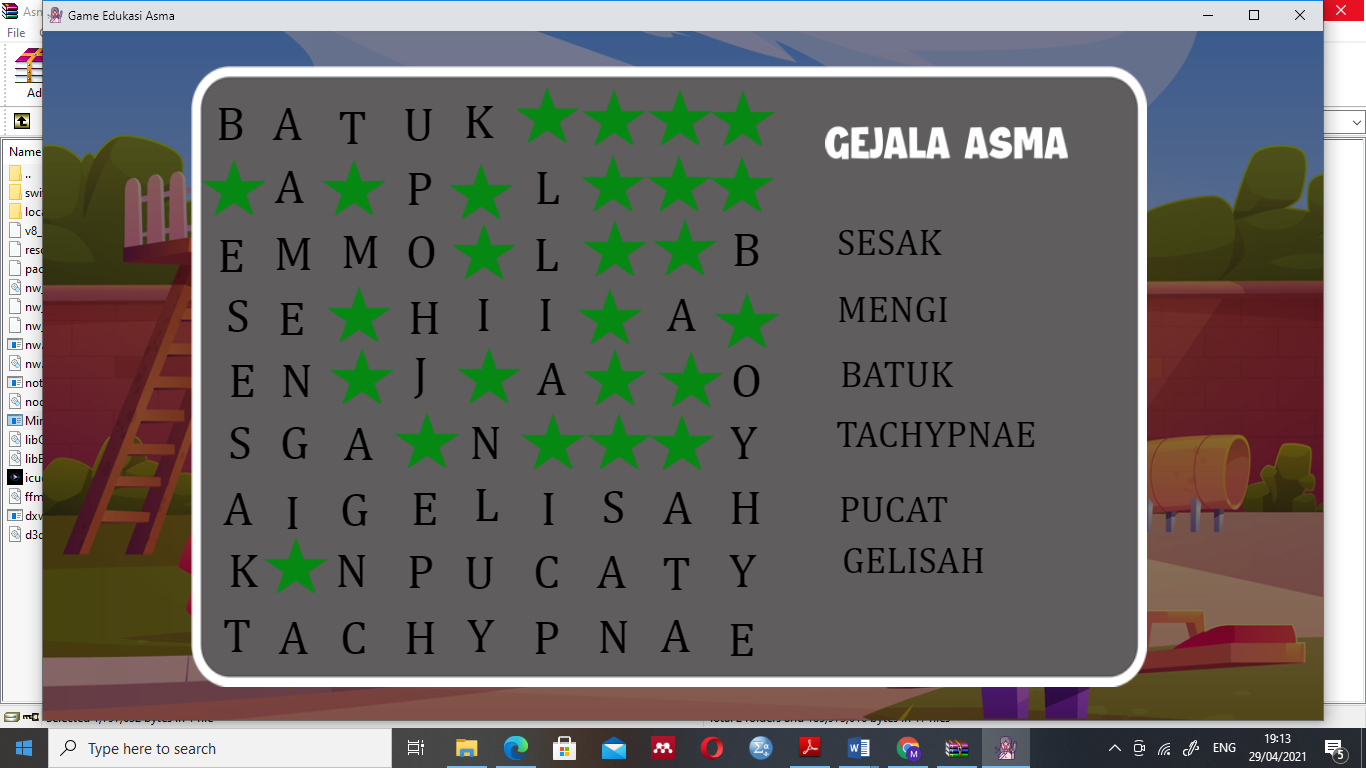 | 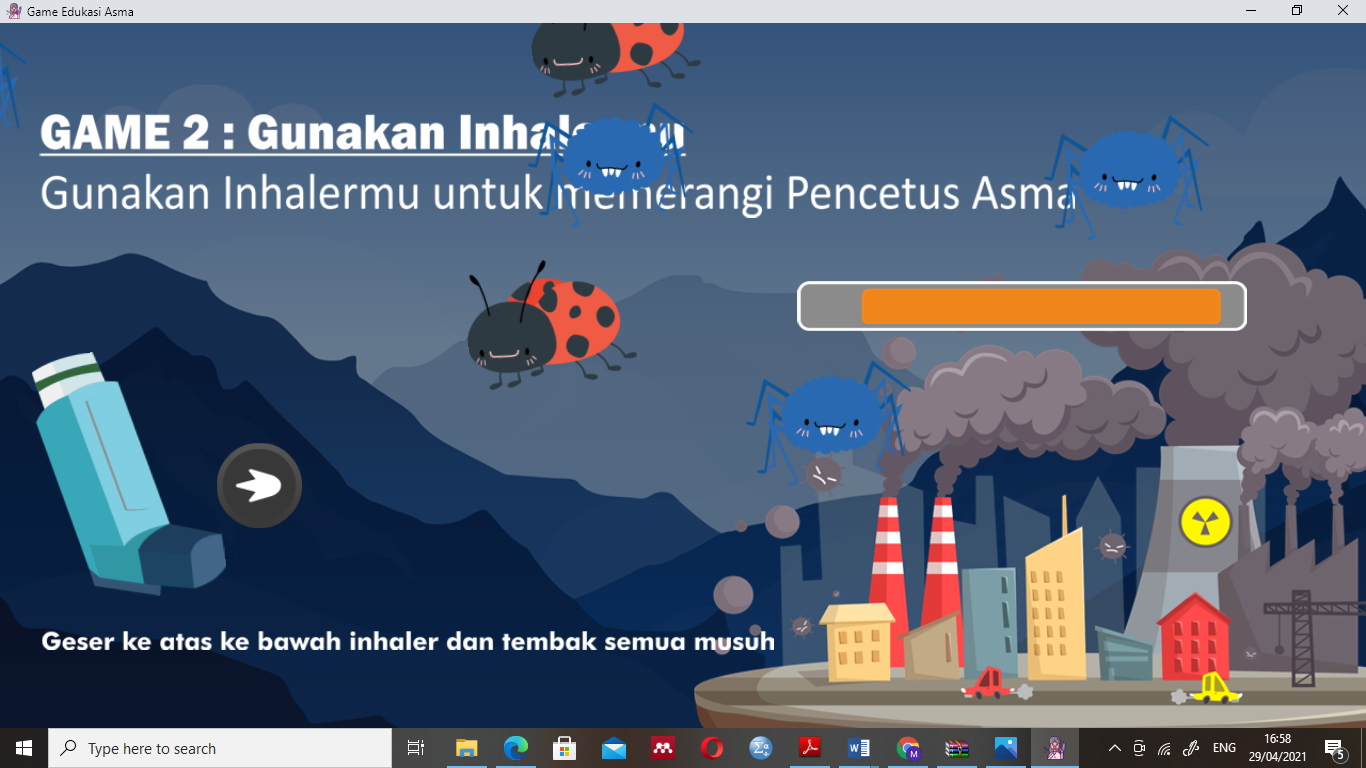 |
| --- | --- |
| 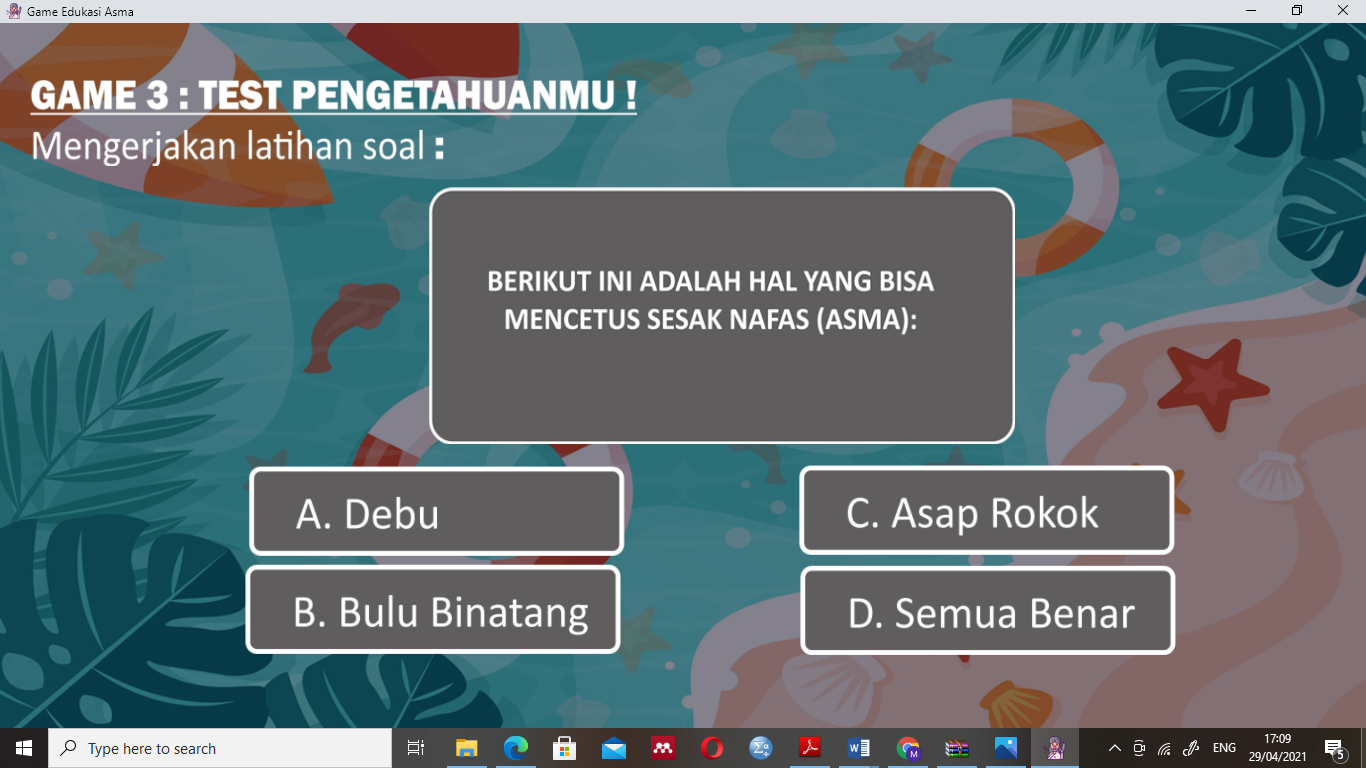 | 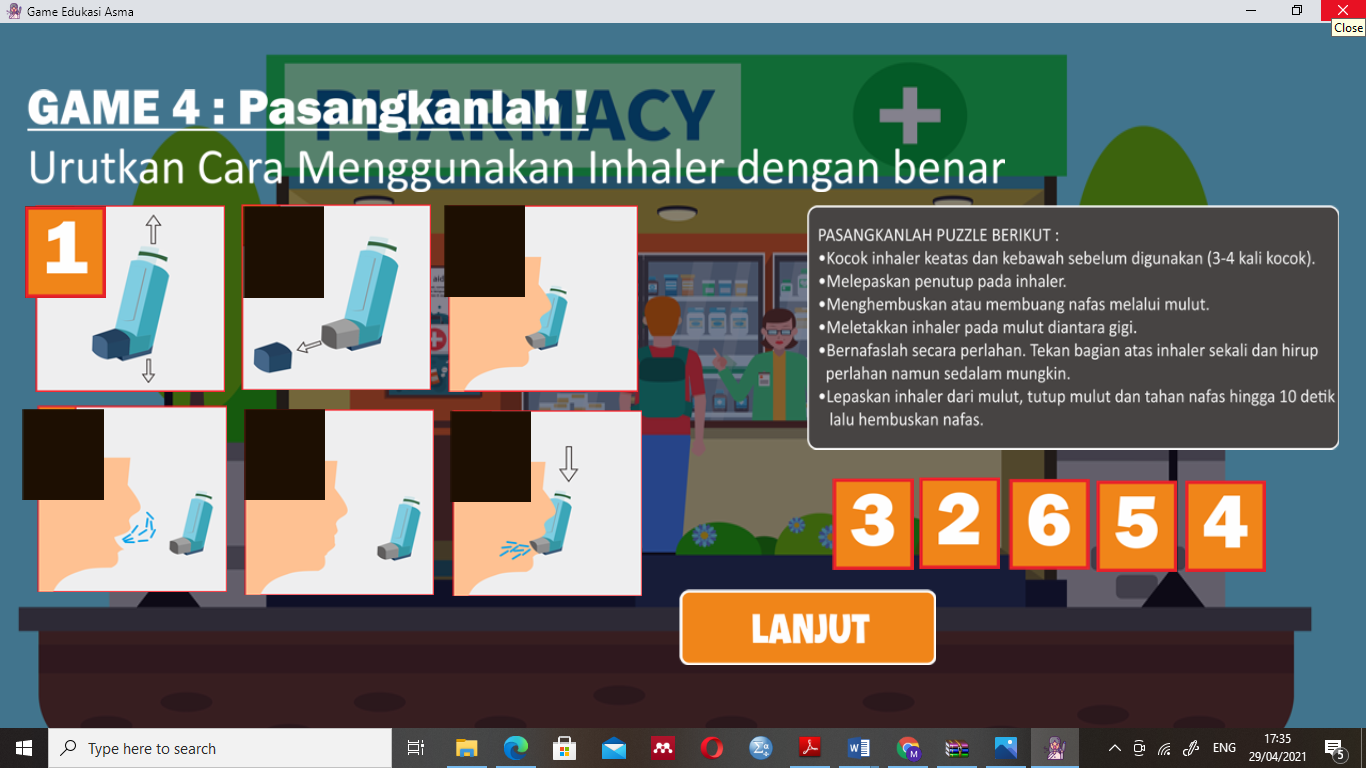 |
| 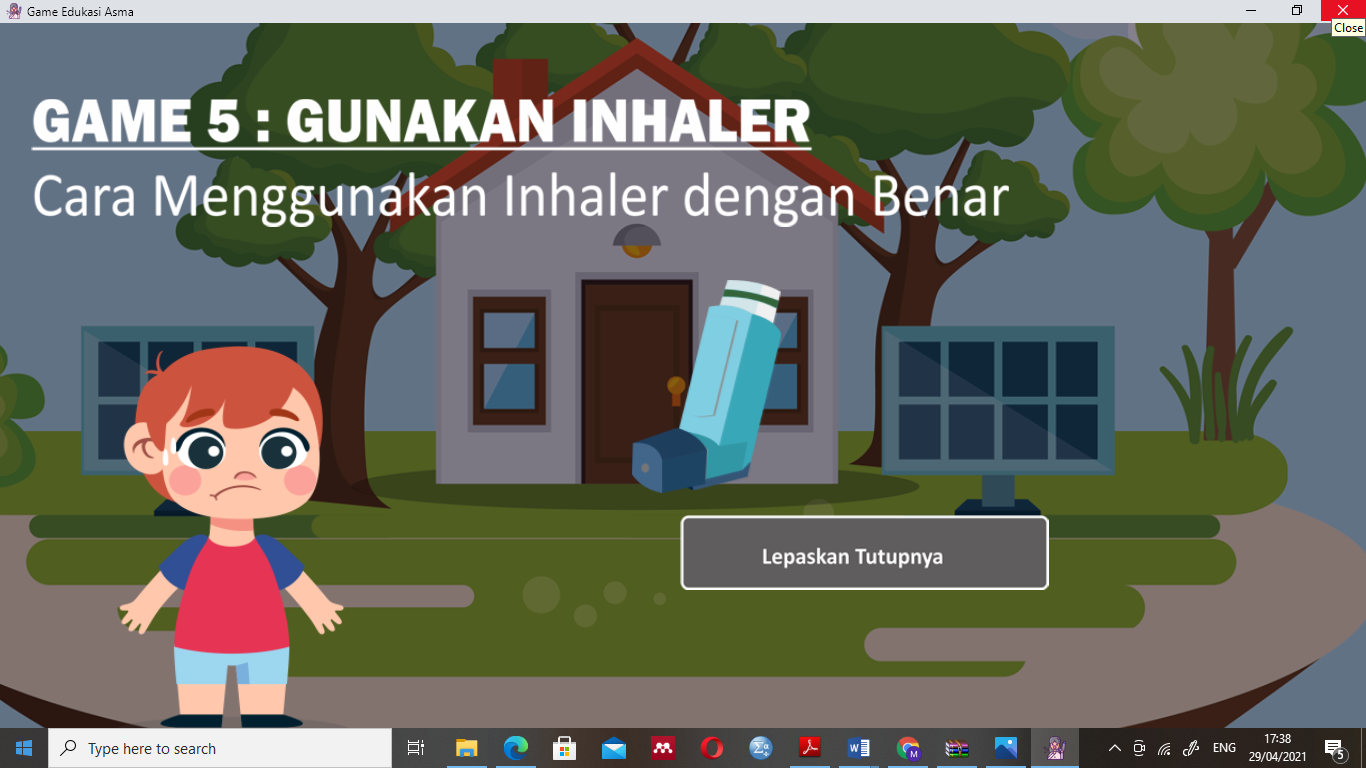 | 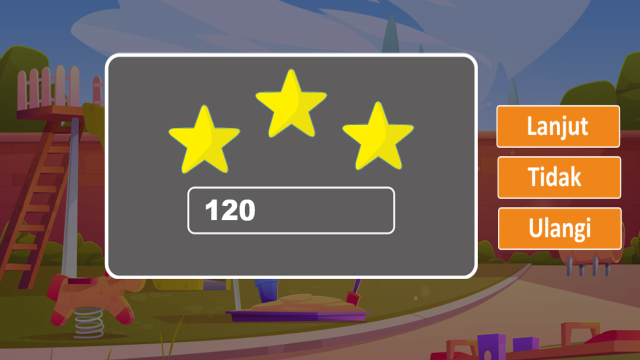 |
