# Supplementary material for: A Computer-Based Interactive Narrative and a Serious Game for Children With Asthma: Development and Content Validity Analysis
Source: J Med Internet Res. 2021 Sep 13;23(9):e28796. doi: 10.2196/28796 (PMC8477291; doi:10.2196/28796)
Supplement: Multimedia Appendix 3 [file jmir_v23i9e28796_app3.docx]

**Multimedia Appendix 3. Elements of asthma self-management and feedback from the experts.**

**Table lists.**

**Table S1.** Elements of asthma self-management……………………………………… 2

**Table S2.** Feedback from the experts………………………………………………….. 4

**Table S1.** The elements of asthma self-management.

| **#** | **Elements of asthma self-management** | **Interactive narrative** | **Serious game** |
| --- | --- | --- | --- |
| 1 | Provide information about normal or healthy airways (lungs). | General information before beginning the plots. | General information before beginning the sessions. |
| 2 | Display information about the condition of the lungs during an asthma attack. | General information before beginning the plots. | General information before beginning the sessions. |
| 3 | Provide information about the definition of asthma. | General information before beginning the plots. | General information before beginning the sessions. |
| 4 | Provide information that an asthma attack can appear anytime and anywhere. | Plots 1, 2, 3, and 4. | Session 2 and 3. |
| 5 | Provide information about asthma triggers. | Plots 1, 2, 3, and 4. | Sessions 2 and 3. |
| 6 | Explain how to avoid things that can trigger asthma attacks. | Plots 1, 2, 3, and 4. | Sessions 2 and 3. |
| 7 | Explain the need to recognize asthma triggers themselves and avoid them. | Plots 1, 2, 3, and 4. | Session 2. |
| 8 | Explain the symptoms felt during an asthma attack. | Plots 1, 2, 3, and 4. | Sessions 1 and 2. |
| 9 | Explain the need to recognize and monitor signs of asthma. | Plots 1, 2, 3, and 4. | Session 1. |
| 10 | Explain to seek help immediately when an asthma attack occurs. | Plots 1, 2, 3, and 4. | Session 5. |
| 11 | Explain to do the following when an asthma attack occurs. | Plots 1, 2, 3, and 4. | Session 5. |
| 12 | Explain uncontrolled symptoms and signs of asthma. | General information before beginning the plots. | General information before beginning the sessions. |
| 13 | Explain the importance of adherence to medications. | Plots 1, 2, 3, and 4. | Sessions 4 and 5. |
| 14 | Explain about asthma treatment in general, for example drug controller and drug reliever. | General information before beginning the plots. | Session 3. |
| 15 | Explain the indications for inhalers; the purpose of inhalers. | General information before beginning the plots. | Session 3. |
| 16 | Explain how to use an inhaler. | Plots 3 and 4. | Sessions 4 and 5. |
| 17 | Explain how to properly clean the inhaler. | Plot 4. | Session 4. |
| 18 | Explain how to correctly store inhalers. | Plot 4. | Session 4. |
| 19 | Explain that asthma is not a contagious disease. | General information before beginning the plots. | General information before beginning the sessions. |
| 20 | Explain that people with asthma can live normally (asthma can be controlled). | General information before beginning the plots. | General information before beginning the sessions. |
| 21 | Explain that asthma drugs do not cause dependence (addiction). | General information before beginning the plots. | General information before beginning the sessions. |
| 22 | Explain the importance of carrying asthma medication when traveling anywhere. | Plots 3 and 4. | Sessions 4 and 5. |
| 23 | Explain that a written action plan is something that needs to be completed | General information before beginning the plots. | General information before beginning the sessions. |
| 24 | Explain the need for regularly scheduled doctor visits. | General information before beginning the plots. | General information before beginning the sessions. |

**Table S2.** Feedback from the experts.

| **#** | **The experts** | **Feedback** |
| --- | --- | --- |
| 1 | Dr. A (pediatrician, Sanglah Hospital, Denpasar, Bali, Indonesia). | *“The content was appropriate for children with asthma, and it emphasized the importance of asthma self-management in daily practice.”* |
| 2 | Pharmacist B, (pharmacist, Kertha Husada Hospital, Singaraja, Bali, Indonesia). | *“The voice should be clearer. Intonation should be more sharpened in critical points. Display, layout, and colorful design were good-looking and represented children's environments”.* |
| 3 | Pharmacist C, (pharmacist/lecturer, Department of Pharmacy, Faculty of Medicine, Brawijaya University, Malang, East Java, Indonesia). | *“Clearly emphasized the proper inhaler technique step by step. The take-home message should be outlined in daily practice, including avoiding asthma triggers, understanding asthma medicine, and understanding how to properly use the inhaler”.* |
